# Astrocyte reactivation in medial prefrontal cortex contributes to obesity-promoted depressive-like behaviors

**DOI:** 10.1186/s12974-022-02529-4

**Published:** 2022-06-27

**Authors:** Gang Yu, Feng Cao, Tingting Hou, Yunsheng Cheng, Benli Jia, Liang Yu, Wanjing Chen, Yanyan Xu, Mingming Chen, Yong Wang

**Affiliations:** 1 Department of Gastrointestinal Surgery, The Second Hospital of Anhui Medical University, Hefei, 230601 China; 2 Bariatric Center, the Second Hospital of Anhui Medical University, Hefei, 230601 China; 3 College of Pharmacy, Sanquan College of Xinxiang Medical University, Xinxiang, 453000 China; 4 Chinese Medicine Modernization and Big Data Research Center, Nanjing Hospital of Chinese Medicine Affiliated to Nanjing University of Chinese Medicine, Nanjing, 210022 China; 5 Department of Neurology, Yale University School of Medicine, New Haven, 06536 USA

**Keywords:** Obesity, High-fat diet, Ob/ob, Chronic social defeated stress, Astrocyte, Stress, Depression, mPFC

## Abstract

**Background:**

Little is known about how the obesogenic environment influences emotional states associated with glial responses and neuronal function. Here, we investigated glial reactivation and neuronal electrophysiological properties in emotion-related brain regions of high-fat diet (HFD) and ob/ob mice under chronic stress.

**Methods:**

The glial reactivation and neuronal activities in emotion-related brain regions were analyzed among normal diet mice (ND), HFD mice, wild-type mice, and ob/ob mice. To further activate or inhibit astrocytes in medial prefrontal cortex (mPFC), we injected astrocytes specific Gq-AAV or Gi-AAV into mPFC and ongoing treated mice with CNO.

**Results:**

The results showed that obesogenic factors per se had no significant effect on neuronal activities in emotion-related brain regions, or on behavioral performance. However, exposure to a chronic stressor profoundly reduced the frequency of spontaneous inhibitory postsynaptic currents (sIPSCs) and spontaneous excitatory postsynaptic currents (sEPSCs) in the mPFC; depressive-like behaviors were seen, accompanied by significant upregulation of astrocyte reactivation. We identified resilient and susceptible mice among chronic social defeat stress-exposed HFD mice. As expected, astrocyte reactivity was upregulated, while neuronal activity was depressed, in the mPFC of susceptible compared to resilient mice. Furthermore, activating astrocytes resulted in similar levels of neuronal activity and depressive-like behaviors between resilient and susceptible mice. Additionally, inhibiting astrocyte reactivation in the mPFC of HFD mice upregulated neuronal activities and inhibited depressive-like behaviors.

**Conclusions:**

These observations indicate that obesogenic factors increase the risk of depression, and improve our understanding of the pathological relationship between obesity and depression.

**Graphical Abstract:**

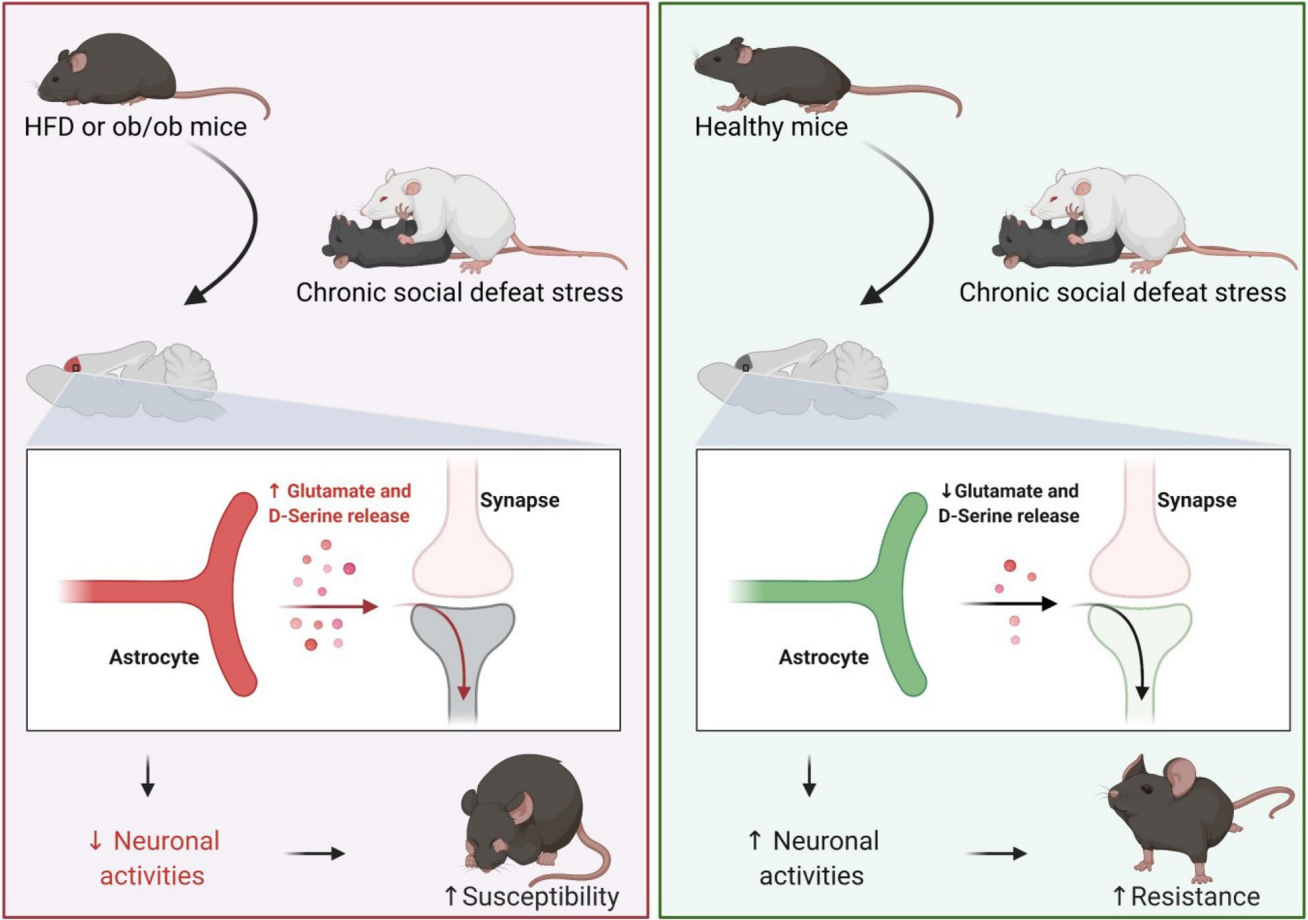

**Supplementary Information:**

The online version contains supplementary material available at 10.1186/s12974-022-02529-4.

## Background

The worldwide prevalence of obesity is exploding; more than 1.9 billion adults and 340 million children and adolescents aged 5–19 years were overweight in 2016, and 39 million children aged < 5 years were overweight in 2020. This represents a serious public health problem. Depression is a major reason for disability and imposes a significant burden on public health systems worldwide [1]. A meta-analysis of 15 longitudinal studies identified a bidirectional relationship between obesity and depression, where obesity and overweight increase the risk of the onset of depression and depression increases the tendency to develop obesity [2]. Obesity in children and adults increases the risk of hypertension, insulin resistance, cardiovascular disease, anxiety, and depression [3, 4], and patients with atypical depression present have a 2.55-fold higher body mass index compared to those with melancholic depression [5]. However, the pathological mechanism is not fully understood.

Environmental and genetic factors are involved in obesity and depression. Prolonged exposure to a high-fat diet (HFD) increases food and caloric intake per meal, leading to weight gain and increased adiposity in humans and animal models; in turn, this contributes to dysregulated energy balance [6]. Moreover, chronic exposure to a HFD induces obesity and increases the levels of tumor necrosis factor and interleukin-6 in amygdaloid nuclei, indicating an inflammatory response in the central nervous system (CNS), as well as enhanced stress reactivity and anxiety-like defensive behavioral responses [7]. Other studies have reported that obesity and depression involve enhanced central inflammation in common brain regions, including the cortices, hippocampus, amygdala and hypothalamus, leading to abnormal neurocircuitry and dysfunction in neurotransmitter systems [8–10]. As a primary participant and contributor on neuroinflammation, astrocytes are subtly controlled and widely respond to both intrinsic and environmental clues [11–14]. To better characterize the molecular feature of reactive astrocytes and inspired by results that only part of genes was well overlapped in response to different stimulation, Barres laboratory defined the pan-, A1- (induced by LPS as well as IL1α, C1q, and TNF and showed toxic effects to neurons and oligodendrocytes), and A2-reactive (showed neuroprotective effects) astrocytes by a set of 10–13 genes [15, 16], which is widely accepted and used in current researches [14]. However, the molecular profile of astrocytes with regard to HFD and obesogenic factors is still elusive.

Astrocytes are glia in the CNS that participate in fundamental processes, including maintenance of the blood–brain barrier, neurovascular and synaptic transmission, energy metabolism, and modulation of neuronal activity [17, 18]. Many studies have revealed that astrocytes are reactivated under chronic neuroinflammatory conditions, such as Alzheimer’s disease and depression [19, 20]. Activated astroglia within the hippocampus become leptin-insensitive following HFD exposure, and leptin-induced modulation of hippocampal synaptic transmission is attenuated under the same conditions [21]. Abnormal neuronal activity and astrocyte reactivation exist in depressed patients and animal models of obesity [22, 23]. Exercise, nutrition control, weight loss and astroglia inhibition alleviate the cognitive decline induced by a HFD [23].

The present sequence of studies assessed the effects of obesity and an HFD on social and emotional behaviors, and neuronal activity, to investigate whether obesity per se affects psychiatric disorders. Neuroinflammation is detectable in the brainstem and hypothalamic neurocircuits responsible for the regulation of food intake and energy homeostasis after exposure to a HFD [24–26], and neuroinflammation-induced dysregulation of neurocircuits and the immunoregulatory cell (microglia and astrocytes) response contribute to synaptic and neuronal dysfunction [27, 28]. Neuronal activity and changes in astrocytes have been detected in obese mice induced by a HFD, and astrocytes are reactivated or inhibited by adeno-associated virus (AAV) in HFD mice. We aimed to determine the pathological mechanism underlying the obesity and depression-like behavior induced by chronic social defeat stress (CSDS).

## Methods

### Animals

The C57BL/6, CD1, and ob/ob mice (leptin-deficient mice in C57BL/6 background) used in this study were purchased from Jackson Laboratories (Bar Harbor, ME, USA). Only male mice were used for study. Littermate (LM) mice were obtained through crossing ob/ob and C57BL/6 mice. All mice were housed at the animal center of Anhui Medical University in a temperature- and humidity-controlled environment, and fed water ad libitum under a 12-h light/12-h dark cycle. All experiments were conducted according to protocols approved by the Institutional Animal Care and Use Committee of Anhui Medical University.

### High-fat diet

All CD1 mice were fed a standard mice chow (10% of calories from fat) throughout the experiment. C57BL/6 mice were randomly assigned on the date of arrival to one of the two diet groups. The HFD consisted of 60% calories, and HFD mice were fed the HFD from day 56 to day 112. The standard diet consisted of 10% calories from fat; the normal diet (ND) mice were fed standard mice chow from day 56 to day 112.

### Chronic social defeat stress

The CD1 mice were isolated for 4 weeks before screening for aggressors. Aggressor CD1 mice were screened via the social interaction (SI) test and used in the CSDS experiment (attack latencies < 30 s on three consecutive screening tests). CSDS was divided into a physical attack phase and sensory contact phase [29]. The ND, HFD, ob/ob, and LM mice were housed with aggressive CD1 mice without a partition for 10 min per day during the physical attack phase. In the sensory contact phase, the ND, HFD, ob/ob, and LM mice were housed with aggressive CD1 mice for 23 h and 50 min per day, but with a perforated Plexiglas divider in the middle of the cage. CSDS was induced repeatedly on 10 consecutive days. Defeated mice were exposed to a new mouse and cage every day.

### Behavioral function testing

The SI and affective behavioral tests, including the SI, open field, elevated plus-maze, tail suspension, and forced swimming tests, were performed according to previous studies [30–34].

The SI test used unfamiliar aggressive CD1 mice enclosed in a wire cage the day after their final defeat session (day 125), or after the final intraperitoneal (i.p.) clozapine-n-oxide (CNO) injection (day 141) was administered as described previously. The SI ratio was calculated as the amount of time that the mouse spent near the enclosure containing the aggressive CD1 mice (social target) over a 2.5-min period compared to when the aggressor was absent. With some modification to previous study, mice with an SI ratio < 1 and TST (tail suspension test) ≥ 100 s were considered “stress-susceptible”, while those with SI ratio ≥ 1 and TST < 100 s were considered “stress-resilient” [35].

The open field and elevated plus-maze tests were used to assess anxiety-like behavior, as in a previous study. The open-field test was performed in an open transparent plastic box (50 cm length × 50 cm width × 40 cm height). Four equal-sized squares with a total area equal to the central square region were marked in the bottom corners of the box. After a 10-min adaptation period, the mice were placed in the corner of the box, and a video recorder was placed on top of the box to record movements for 10 min. The time spent in the central area was quantified by the VisuTrack system (Xinruan Information Technology Co., Ltd., Shanghai, China). The elevated plus-maze contained two closed arms (30 cm) enclosed by 14-cm-high walls and two open arms (30 cm). The arms were extended from a 5-cm square platform, and the maze was 45.5 cm from the floor. The mice were placed on the central platform and permitted to explore the plus-maze. Motion paths were video-recorded for 10 min from the top of the plus-maze. The time spent in the open arms was analyzed by the VisuTrack system.

The tail suspension, forced swimming, and sucrose preference tests were conducted to assess depression-like behaviors. For the tail suspension test, the tails of the mice were cleaned and adhered to “stoppers” using adhesive tape (at approximately half the distance from the base) to prevent climbing; a clip was then attached to the adhesive tape. Mice were suspended 20–30 cm above the floor by the clip and videotaped for 6 min. The immobility time (during the last 4 min of the 6-min test), i.e., the time during which the mice did not attempt to move their limbs and remained in a vertical posture during suspension, was analyzed by ANY-maze software (Stoelting, Kiel, WI, USA). In the forced swimming test, a mouse was placed in a 40-cm-high and 18-cm-diameter Plexiglas cylinder filled with 15 cm of water (23–25 °C). The mouse swam in the cylinder for 6 min. Movements were recorded and the immobility time was analyzed by ANY-maze software. For the sucrose preference test, mice were habituated alone for 24 h in the behavioral testing room with water. The mice were provided with one plain water bottle and one water bottle with 1% sucrose solution for the next 2 days. Daily water and sucrose intake were quantified on the last day, and sucrose preference was calculated as the volume of sucrose intake over the volume of total fluid intake.

### Adipose tissue mass

Adipose tissues were obtained as described in a previous study [36]. In brief, mice were killed, and epididymal fat tissues (white fat) were separated from the mouse testis. Retroperitoneal tissues were isolated from perinephric white adipose fat. Inguinal fat tissues were defined as white fat tissues located in the inguinal region.

### Electrophysiological analysis

Patch-clamp whole-cell recording was applied to neurons in the hippocampus, amygdala, and medial prefrontal cortex (mPFC), and inhibitory postsynaptic currents (IPSCs) and excitatory postsynaptic currents (EPSCs) were measured and analyzed according to the protocol used in a previous study [37]. In brief, 300-µm-thick brain slices were obtained and cultivated in 4 °C oxygenated high-sucrose artificial cerebrospinal fluid (aCSF) (95% O_2_/5% CO_2_ over 2 h; 87 mM NaCl, 2.5 mM KCl, 7 mM MgCl_2_, 1.25 mM NaH_2_PO_4_, 25 mM NaHCO_3_, 25 mM glucose, and 75 mM sucrose, pH 7.3). Brain slices were recovered in a culture chamber filled with oxygenated aCSF (95% O_2_/5% CO_2_ over 2 h; 124 mM NaCl, 3 mM KCl, 1 mM MgCl_2_, 1.25 mM NaH_2_PO_4_, 26 mMNaHCO_3_, and 10 mM glucose, pH 7.3), and the temperature was maintained at 32–34 °C for this step. The brain slices were transferred to a recording chamber perfused with aCSF, and the micropipettes were filled with working buffer (1 mM MgCl_2_, 0.2 mM EGTA, 4 mM Mg-ATP, 0.3 mM Na-GTP, 125 mM Cs-methanesulfonate, 5 mM CsCl, 10 mM phosphocreatine, and 5 mM QX314; pH 7.3, 285 mOsm). EPSCs and IPSCs of the neurons in the hippocampus, amygdala, and mPFC were recorded via a micropipette and an upright microscope equipped with a 40 × water-immersion lens (Axioskop 2 Plus; Zeiss, Oberkochen, Germany). Spontaneous excitatory postsynaptic currents (sEPSCs) and spontaneous inhibitory postsynaptic currents (sIPSCs) were recorded with the MultiClamp 700B and 1440A digitizer (Molecular Devices, Sunnyvale, CA, USA). Miniature excitatory postsynaptic currents (mEPSCs) and miniature inhibitory postsynaptic currents (mIPSCs) were recorded after 1 µM TTX was administered (MCE, Suzhou, China).

### Magnetic-activated cell-sorting and flow cytometry

mPFC tissues were isolated and digested with 300 µg/mL DNase I (Sigma, St. Louis, MO, USA) and 1 mg/mL papain (MCE) for 30 min at room temperature. The cell pellets were collected by centrifugation at 300×*g* for 10 min, and astrocytes and microglia were isolated according to the manufacturer’s instructions for the anti-GLAST Microbeads and CD11b-Microbeads (Miltenyi Biotech, Gladbach, Germany). The microglia and astrocyte cell pellets were suspended in 1 × phosphate-buffered saline (PBS). The astrocytes were incubated with anti-GLAST-APC (Miltenyi Biotec), and the microglia were incubated with anti-CD45-APC and anti-CD11b-pe (both from Miltenyi Biotec). The flow cytometry assay was performed with the Attune NxT system (BD Biosciences, San Jose, CA, USA).

### Quantitative real-time polymerase chain reaction (qRT-PCR)

Total RNA was extracted from isolated microglia, astrocytes, or mPFC tissues using TRIzol reagent (Invitrogen, Carlsbad, CA, USA). Sample contents were quantified using the NanoDrop spectrophotometer (Thermo Fisher, Waltham, MA, USA). cDNA was obtained by RNA reverse transcription amplification from total RNA using the Revert Aid First Strand cDNA Synthesis kit (Thermo Scientific). The qRT-PCR was conducted with SYBR Green Real-time PCR Master Mix (Toyobo, Shiga, Japan). Leptin mRNA expression was analyzed using the CFX96 Real-time PCR System (Bio-Rad Laboratories, Hercules, CA, USA) and determined by the 2^−ΔΔCt^ method. The used primers are listed in Additional file 1: Table S1.

### Immunofluorescence staining

Mice were anesthetized with ketamine (85 mg/kg) and perfused through the aorta with 1 × PBS, followed by 4% paraformaldehyde (PFA). Brain tissues were dissected and immersed in 4% PFA for 24 h and then dehydrated in 20% sucrose for 3 days. The dehydrated brain tissues were frozen and sectioned using a cryostat (CM1900; Leica, Wetzlar, Germany). Consecutive coronal section (10 µm) containing the mPFC were obtained, fixed in 4% PFA for 5 min, washed in 1 × PBS, and blocked in 10% horse serum containing 0.1% Triton X-100 in 1 × PBS for 1 h at room temperature. After blocking, the sections were washed in 1 × PBS and incubated with an antibody against GFAP (1:1000) (DAKO, Santa Clara, CA, USA) at 4 °C overnight. The sections were then washed in 1 × PBS and incubated with Alexa Fluor 488 polyclonal antibody (Thermo Fisher) for 1 h at room temperature. Then, the slices were washed in 1 × PBS and sealed with SlowFade gold mountant (Thermo Fisher). GFAP + astrocytes were randomly selected from the mPFC, and images were obtained using a confocal microscope (FV1200; Olympus, Tokyo, Japan) and analyzed with ImageJ software (NIH, Bethesda, MD, USA).

### Western blot

Fresh samples were extracted and homogenized with radioimmunoprecipitation assay buffer (Biosharp, Hefei, China) on ice containing phenylmethanesulfonyl fluoride (Biosharp) and phosphatase inhibitors (Roche, Basel, Switzerland). The protein concentration was quantified with the BCA Protein Quantitation Kit (Thermo Scientific). The proteins were denatured at 95 °C for 5 min and diluted with 5 × loading buffer. The protein samples were separated by 10% sodium dodecyl sulfate-polyacrylamide gel electrophoresis and transferred to a polyvinylidene fluoride membrane (Millipore, Paisley, UK). The membranes were blocked with 5% BSA at room temperature for 2 h and then incubated with primary leptin antibody (1:1000; Abcam, Cambridge, UK). The membranes were washed in Tris-buffered saline containing 0.1% Tween-20 and incubated with an HRP-conjugated secondary antibody for 2 h (1:5000) (Proteintech, Rocky Hill, NJ, USA) at room temperature. The band densities were analyzed by ImageJ software and GADPH (1:5000; Proteintech) was used as the control.

### AAV stereotaxic injection and CNO treatment

Mice were anesthetized and fixed in a stereotaxic apparatus as described previously [37]. A hole was drilled based on the position of the bregma, and 0.3 µl of AAV was delivered via a Hamilton syringe (mPFC; AP = + 1.96 mm, ML = ± 0.42 mm, DV = − 1.62 mm) at a speed of 0.1 µl/min. The injection needle was withdrawn 10 min after injection. AAV2/9-GfaABC1D- hM3Dq-mCherry-WPRE-pA (titer, 1.76 × 10^13^ vg/mL), AAV2/9-GfaABC1D-hM4Di-mCherry-WPRE-pA (titer, 1.72 × 10^13^ vg/mL) and the corresponding AAV control were purchased from HANBIO Technology Co. (Shanghai, China). CNO (MCE) or saline was i.p.-injected once per day at a dose of 1 mg/kg/day.

### Microdialysis

Mice were deeply anesthetized and fixed in a stereotaxic apparatus. A guide cannula was implanted into the mPFC and then a microdialysis probe was mounted inserted through the guide cannula and mounted to a CMA microinjection pump to continuously perfuse aCSF at a constant flow rate of 1 µl/min. Samples were collected with a microfraction collector. D-serine concentrations were measured by high-performance liquid chromatography (HPLC) as described previously [38]. ATP, glutamate, and GABA levels were quantified with the ATP Bioluminescence Assay Kit (Roche, Mannheim, Germany), glutamate colorimetric assay kit (BioVision, Milpitas, CA, USA), and GABA ELISA kit (LSBio, Seattle, WA, USA) according to the manufacturer’s instructions.

## Multiplexed phospho-protein and cytokine signaling analysis

To investigate the potential mechanisms, we lysed isolated astrocytes and abstracted total protein by RIPA buffer (Thermo Scientific). After measuring protein concentration by Nanodrop (Thermo Scientific), we detected multiple protein expression level by loading equal amount of total protein with the Milliplex MAP Kit (MilliporeSigma) according to the manufacturer’s instructions.

### Statistical analysis

All data are presented as mean ± standard error and were analyzed using GraphPad Prism 8 (GraphPad Software Inc., La Jolla, CA, USA). One-way ANOVA analysis of variance was conducted to assess the interactions among the ND, HFD, LM, and ob/ob mice, and Bonferroni’s post hoc analysis was performed as appropriate. The unpaired two-tailed Student’s *t*-test was performed to compare two groups or treatments. *p*-values < 0.05 were considered significant.

## Results

### HFD and leptin knockout increase depression-like behavior under 10-day CSDS

Exposure to stress induces depressive-like behaviors in male and female animal models [39, 40]. To avoid the bias induced by individual differences, we excluded c57BL/6 mice with normal social functioning on the social interaction experiment whose SI ratios were > 0.2. Anxious mice were excluded based on the open field and elevated plus-maze tests, as animals that spent < 20 s in the central zone and open arms (Additional file 1: Fig. S1A). Mice without congenital depressive-like behavior whose immobility times on the tail suspension and forced swimming tests exceeded 20 s were used for the next experiment (Additional file 1: Fig. S1A). Leptin mRNA and protein levels were significantly downregulated in ob/ob mice compared to LM mice, as identified by PCR and western blot (Additional file 1: Fig. S1B). As expected, body weight (BW) on day 125/BW on day 56, epididymal fat, retroperitoneal fat, and inguinal fat increased in the HFD and ob/ob mice compared to the ND and LM mice, suggesting that a fat-dense diet and leptin knockout induced changes in energy metabolism (Additional file 1: Fig. S1C). Consumption of the HFD and downregulation of leptin in ob/ob mice did not significantly affect social behavior, as reflected by the SI ratio on the SI test and anxiety-like behaviors (based on the time spent in the central zone of the open-field test and arms of the elevated plus-maze test; Additional file 1: Fig. S1D–H). Although immobility time on the forced swimming test, immobility on the open-field test, and sucrose consumption on the sucrose preference test did not vary among ND, HFD, LM, and ob/ob mice, immobility time on the tail suspension test increased in ob/ob mice compared to ND and LM mice, indicating that an obesogenic factor augmented the tendency toward depressive-like behavior.

**Fig. 1:**
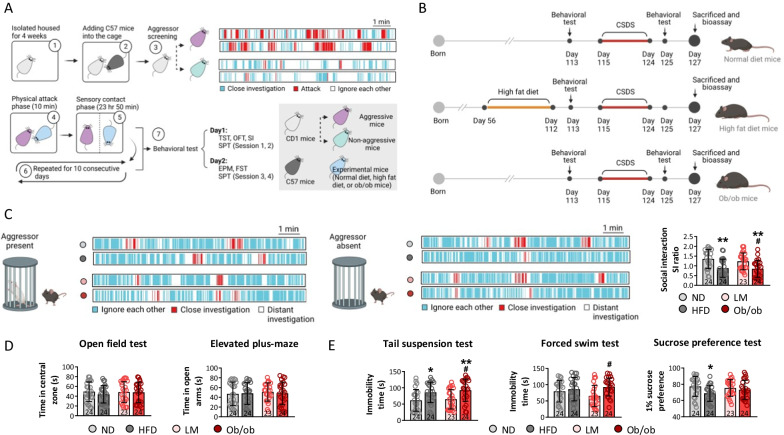
Both high-fat diet and leptin knockout significant aggravated depressive-like behaviors post-chronic stress exposure. Diagram of CSDS model mice establishment (**A**) and experimental design (**B**). To assess the behavioral changes among different mice model, social interaction (**C**) was performed under aggressor present and absent. **D** The anxiety behaviors of mice were evaluated by open-field test and elevated plus-maze, and **E** depressive behaviors of mice were investigated by tail suspension test, forced swim test, and sucrose preference test. (ND represented as normal diet, HFD represented as high-fat diet, LM represented as littermate mice, and ob/ob represented as obesity transgenic mice; *p < 0.05, ***p < 0.001 compared with ND mice; ^#^*p* < 0.05 compared with LM mice)

To conduct the CSDS stimulation, experimental mice were housed with aggressive CD1 mice, as identified by the SI test, for 10 consecutive days with a 10-min physical attack phase. Behavioral tests were performed the next day (Fig. 1A). HFD mice were fed an energy-dense diet (> 60% of calories from fat) from day 56 to day 112. The other experimental schedules are shown in Fig. 1B. Furthermore, social behavior was assessed based on the SI ratio, defined as the time that the mice spent closely investigating the enclosure with the aggressor absent compared to when the aggressor was present. HFD and leptin knockout decreased social behavior, as reflected by a decrease in the SI ratio of the HFD and ob/ob mice compared to ND mice. The SI ratio of ob/ob mice was lower than that of LM mice (*p* < 0.05) (Fig. 1C). No marked differences in the time spent in the central zone in the open-field test, or in the open arm in the elevated plus-maze, were observed among the ND, HFD, LM, and ob/ob mice (*p* > 0.05), suggesting that the HFD and the obesogenic environment did not alter anxiety-like behaviors (Fig. 1D). The tail suspension, forced swimming, and sucrose preference tests were carried out to evaluate depressive-like behaviors in the ND, HFD, LM, and ob/ob mice after CSDS. The HFD increased immobility time on the tail suspension test and decreased sucrose preference by 1% on the sucrose preference test compared to the ND, indicating that an energy-dense diet induced depressive-like behaviors after CSDS stimulation (Fig. 1E). The immobility times of ob/ob mice on the tail suspension test and forced swimming test were longer than those of LM mice, indicating that the obesogenic environment induced depressive-like behavior after CSDS stimulation (Fig. 1E). Taken together, these findings suggest that the HFD and leptin knockout decreased social behavior and induced depressive-like behavior under CSDS conditions.

### HFD and leptin knockout decrease neuronal activity and dendritic spine density in the mPFC under CSDS conditions

To investigate the regulatory roles of the HFD and obesogenic factors in excitatory and inhibitory neuronal activities in brain areas related to emotion regulation, spontaneous action potentials and miniature synaptic potentials were recorded in the hippocampus and amygdala using whole-cell voltage-clamp recording (Additional file 1: Fig. S2). The behavior of the mEPSCs was measured by blocking sodium channel-mediated action potential activity with TTX, and mIPSCs were detected by blocking action potentials and glutamatergic inputs with TTX. No significant differences in the frequency or amplitude of sIPSCs, sEPSCs, mIPSCs, or mEPSCs were observed in the hippocampus or amygdala among the ND, HFD, LM, and ob/ob mice, suggesting that the HFD and leptin knockout did not alter excitatory or inhibitory neuronal activities and astrocytes reactivation in the hippocampus or amygdala under CSDS conditions (Additional file 1: Fig. S2A–N).

**Fig. 2:**
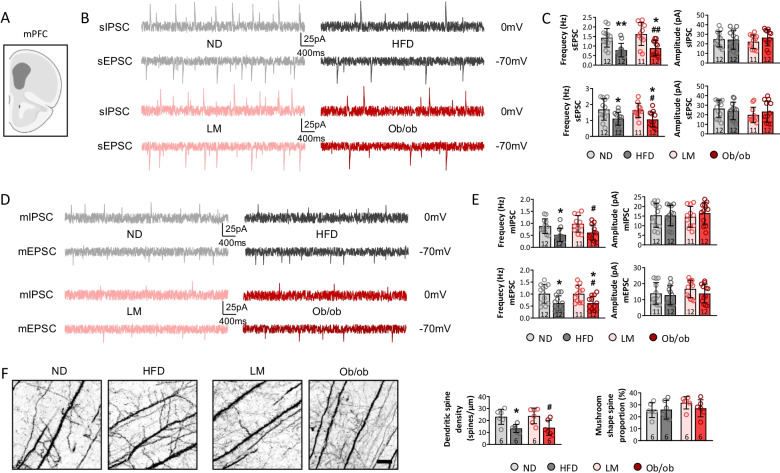
Neuronal activity and synaptic plasticity in mPFC were significantly depressed in high-fat diet and leptin knockout mice. To detect neuronal activities in mPFC (**A**), sIPSC, sEPSC (**B**, **C**), mIPSC, and mEPSC (**D**, **E**) were recorded, and frequency and amplitude were calculated. Additionally, synaptic morphology was assessed by calculating dendritic spine density and mushroom shape spine proportion (**F**). (*p < 0.05, **p < 0.01 compared with ND mice; ^#^*p* < 0.05, ^##^*p* < 0.01 compared with LM mice)

The mPFC is implicated in emotional processing in primates, and evidence shows that abnormal circuitry in the mPFC and hippocampus is associated with depressive behavior [41, 42]. Spontaneous and miniature synaptic potentials were examined in the mPFC after CSDS in this study. The HFD and leptin knockout decreased the frequency of sIPSCs and sEPSC compared to the ND, and a lower frequency of sIPSCs and sEPSCs was observed in ob/ob mice than LM mice (*p* < 0.05) (Fig. 2A–C), indicating that an energy-dense diet and obesogenic factors inhibited excitatory and inhibitory neuronal activities in the mPFC. mIPSCs and mEPSCs were recorded in the four groups to investigate whether presynaptic or postsynaptic release affected neuronal activity in the mPFC. The results showed that the frequencies of mIPSCs and mEPSCs visibly decreased in the HFD and ob/ob mice compared to the ND mice, and the frequency of mIPSCs and mEPSCs in ob/ob mice decreased compared to LM mice (*p* < 0.05) (Fig. 2D and E). No marked changes in the amplitudes of the sIPSCs, sEPSCs, mIPSCs, or mEPSCs were observed among the groups (*p* > 0.05) (Fig. 2C and E), indicating that presynaptic release was inhibited by the energy-dense diet and leptin knockout.

Dendritic spines are the foundation of information transmission and neuroplasticity, and their mushroom-like morphology is functional [43]. To explore the synaptic changes in detail, Golgi staining of the mPFC of the mice was performed. The findings showed that the HFD and leptin knockout decreased dendritic spine density compared to the ND and LM mice, while the proportion of mushroom-shaped spines was not different among the four groups (Fig. 2F). Thus, the energy-dense diet and obesogenic environment suppressed synaptic transmission, thereby inhibiting neuronal excitation and inhibitory activity, and enhancing CSDS-induced depressive-like behaviors. A previous research showed that the E/I balance in the mPFC is important for social behaviors [44], however, E/I ratio was slightly increased in HFD and ob/ob mice, no significance was observed among those groups (Additional file 1: Fig. S3A and B).

### HFD and obesity activate astrocytes and increase the concentration of D-serine and glutamate under CSDS conditions

Postmortem brain tissues of suicide victims who were in a depressive episode indicate enhanced astrocyte and microglia activation, which is associated with aberrant inflammatory processes [45]. In this study, microglia and astrocytes were isolated from mPFC tissues, and mRNA expression levels (reflecting different glial phenotypes) were determined to test the effect of the HFD and leptin knockout on microglia and astrocytes after CSDS. Microglia are influenced by exogenous and endogenous factors, and their phenotypes have been divided into M1, M2a, M2b, and M2c based on various secreted inflammatory factors [46]. The mRNA levels of cellular factors expressing the M1, M2a, M2b, and M2c microglial phenotypes were inspected and the data showed no differences among the ND, HFD, LM, and ob/ob mice after the 10-day CSDS (Additional file 1: Fig. S4A and B). However, we detected the phenotypes, including Pan-reactive, A1-specific, and A2-specific, and mRNA expression of the Pan-reactive and A1-specific markers increased in the HFD and ob/ob mice compared to the ND and LM mice after the 10-day CSDS, showing that HFD and leptin knockout induced neurotoxic astrocytic reactivation (Additional file 1: Fig. S4C and D).

**Fig. 3:**
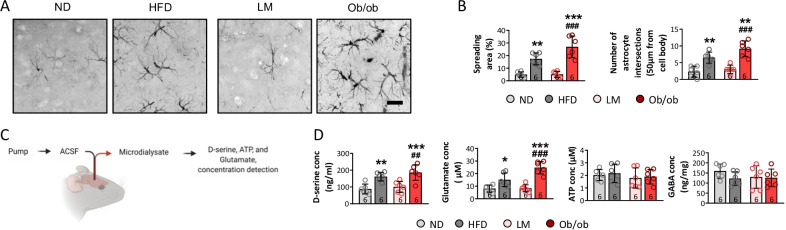
High-fat diet and leptin knockout dramatically increase astrocyte reactivation. **A** Astrocyte morphology was measured by calculating (**B**) spreading area and number of interactions. The level of D-serine, glutamate, ATP, and GABA were also assessed in microdialysate from mPFC (**C** and **D**). (*p < 0.05, **p < 0.01, ***p < 0.001 compared with ND mice; ^##^*p* < 0.01, ^###^*p* < 0.001 compared with LM mice)

To further confirm the activation of astrocytes, the morphology and number of astrocytes in the mPFC were tested by GFAP immunohistochemistry (Fig. 3A). The results showed that the spreading area and number of intersecting astrocytes increased in response to the HFD and leptin knockout compared to ND mice. Moreover, the spreading area and number of intersecting astrocytes increased in ob/ob mice compared to LM mice after CSDS (Fig. 3B), indicating that the HFD and leptin knockout reactivated more astrocytes in the mPFC. In addition, the microdialysate was obtained by a pump placed in the lateral ventricle, and concentrations of D-serine, ATP, glutamate, and GABA involved in astrocyte functions were detected via HPLC and the ATP bioluminescence assay after CSDS (Fig. 3C). As expected, the concentrations of D-serine and glutamate increased in HFD and ob/ob mice compared to ND mice. Furthermore, higher concentrations of D-serine and glutamate were seen in ob/ob than LM mice, consistent with the change in astrocyte morphology (Fig. 3D). These findings suggest that the HFD and leptin knockout increased astrocytic neurotoxic reactivation, which was confirmed by changes in morphology, mRNA expression, and functional release rather than the promotion of astrocytic proliferation after CSDS.

### Activation of astrocytes results in similar neuronal activity and depressive-like behaviors between resilient and susceptible mice

To precisely determine the effects of astrocytes on social and depressive-like behaviors in HFD and ob/ob mice subjected to CSDS, resilient and susceptible mice were identified by the procedures shown in Additional file 1: Fig. S4A. Resilient mice were assumed to have an SI ratio ≥ 1 and immobility time < 100 s, whereas susceptible mice had an SI ratio < 1 and immobility time ≥ 100 s (Additional file 1: Fig. S5A and B). BW on day 140/BW on day 56, and the epididymal, retroperitoneal, and inguinal fat masses, did not differ significantly between resilient and susceptible mice (Additional file 1: Fig. S5C), suggesting that resilience/susceptibility does not influence body development in HFD mice after CSDS. Interestingly, the expression of all astrocytic phenotypes, including pan-reactive, A1-specific, and A2-specific, increased, indicating reactivation of astrocytes (Additional file 1: Fig. S5D). The morphology of the astrocytes in the mPFC was observed to assess astrocytic reactivation, and the astrocytic spreading area increased significantly in susceptible compared to resilient mice (*p* < 0.05), while the number of astrocyte intersections tended to increase (*p* = 0.068) (Additional file 1: Fig. S5E). The concentrations of D-serine, glutamate, ATP, and GABA were measured in the microdialysate, and glutamate release increased in susceptible compared to resilient mice (Additional file 1: Fig. S5F), indicating additional astrocytic reactivation in susceptible HFD mice after CSDS. Additionally, sIPSCs and sEPSCs were detected via whole-cell patch-clamp to investigate neuronal activities. The frequencies and amplitudes of the sIPSCs and sEPSCs decreased in susceptible compared to resilient mice (Additional file 1: Fig. S5G-I).

**Fig. 4:**
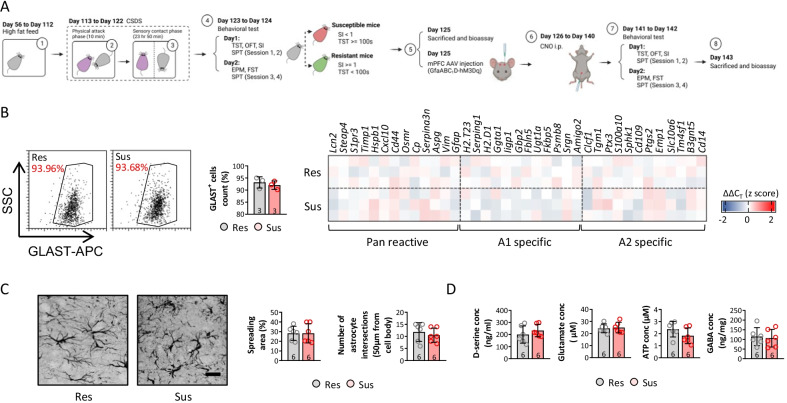
mPFC injection of GfaABC1D-hM3Dq-AAV and CNO i.p treatment significantly upregulated astrocytes reactivity. Diagram of experimental design (**A**). Astrocyte response was detected by investigating activation related genes expression of isolated astrocyte (**B**). Astrocyte morphology was measured by calculating (**C**) spreading area and number of interactions. The level of D-serine, glutamate, ATP, and GABA were also assessed in microdialysate from mPFC (**D**). (*p* < 0.05 was considered as significant difference)

As GfaABC1D-hM3Dq-AAV following i.p. injection of CNO can induce the reactivation of astrocytes, we performed the procedures shown in Fig. 4A, including an additional mPFC GfaABC1D-hM3Dq-AAV injection on day 125 and i.p. injections of CNO from day 126 to day 140, to enhance astrocytic reactivation in the mPFC (Fig. 4A). GLAST + cells were isolated from the mPFC and the mRNA levels of associated astrocyte phenotype markers were similar between susceptible and resilient mice (Fig. 4B), confirming astrocytic reactivation via the GfaABC1D-hM3Dq-AAV and CNO i.p injections. Consistent with the change in the number and phenotype of mPFC astrocytes, the spreading area and number of astrocyte intersections identified by the morphological examination were not different between susceptible and resilient mice after astrocytic activation (Fig. 4C). The concentrations of D-serine, glutamate, ATP, and GABA in the microdialysate were similar between susceptible and resilient mice, as expected (Fig. 4D). These findings suggest that the mPFC GfaABC1D-hM3Dq-AAV and CNO i.p. injections reactivated astrocytes, accompanied by phenotypic, morphological, and release changes in susceptible and resilient mice.

**Fig. 5:**
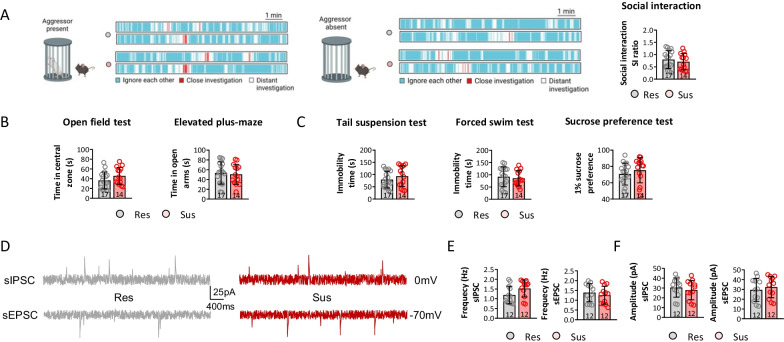
Activating astrocytes in mPFC of resilient mice induces similar depressive-like behaviors compared with susceptible mice. **A** To assess the behavioral changes among different mice model, social interaction was performed under aggressor present and absent. **B** The anxiety behaviors of mice were evaluated by open-field test and elevated plus-maze, and **C** depressive behaviors of mice were investigated by tail suspension test, forced swim test, and sucrose preference test. To detect neuronal activities in mPFC, sIPSC and sEPSC (**D**) were recorded, and frequency and amplitude were calculated (**E** and **F**). (*p* < 0.05 was considered as significant difference)

Given the difference in astrocyte reactivation between susceptible and resilient mice after the GfaABC1D-hM3Dq-AAV and CNO i.p. injections, behavioral examinations were performed. Data showed a significant decrease of SI ratio (Additional file 1: Fig. S6A) and increase of immobility time on tail suspension and forced swim test (Additional file 1: Fig. S6B-D), together with a dramatic decrease of sIPSC and sEPSC frequencies in susceptible mice (Additional file 1: Fig. S6E-G), when comparing with resilient mice, before hM3Dq-CNO administration. In contrast, no differences in the SI ratio, time spent in the central zone, time spent in the open arms, immobility time, or 1% sucrose preference were observed between susceptible and resilient mice after the GfaABC1D-hM3Dq-AAV and CNO i.p. injections (Fig. 5A–C). Furthermore, sIPSCs and sEPSCs were detected after the mPFC GfaABC1D-hM3Dq-AAV and CNO i.p. injections. The susceptible and resilient mice showed similar sIPSC and sEPSC frequencies and amplitudes (Fig. 5D–F). These findings indicate that increased astrocytic reactivation led to similar neuronal activities, in turn contributing to similar social and depressive-like behaviors between susceptible and resilient mice.

### Inhibiting astrocytes reduces the frequencies of sIPSCs and sEPSCs, and increases depressive-like behaviors in susceptible mice

**Fig. 6:**
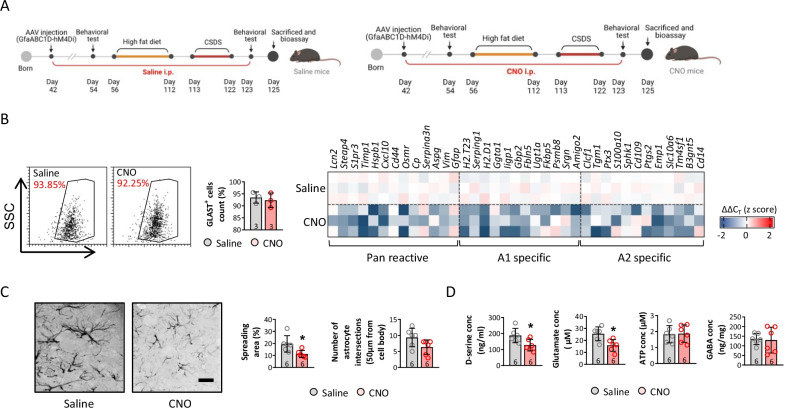
mPFC injection of GfaABC1D-hM4Di-AAV and CNO i.p treatment significantly inhibited astrocytes reactivity. Diagram of experimental design (**A**). Astrocyte response was detected by investigating activation related genes expression of isolated astrocyte (**B**). Astrocyte morphology was measured by calculating (**C**) spreading area and number of interactions. The level of D-serine, glutamate, ATP, and GABA were also assessed in microdialysate from mPFC (**D**). (*p < 0.05 compared with saline-treated mice)

To confirm the role of astrocytic reactivation in obesity-related depressive-like behavior and inhibited neuronal activity, mice with normal social function were identified based on an SI ratio > 0.1 and < 2. Mice with anxiety- and depressive-like behaviors were excluded using the open field, elevated plus-maze, tail suspension, and forced swimming tests (Additional file 1: Fig. S7A). The mice performed the tests according to the schedule shown in Fig. 6A schedule, and activation of mPFC astrocytes was inhibited by the mPFC GfaABC1D-hM4Di AAV and CNO i.p. injections (Fig. 6A). Interestingly, activation of mPFC astrocytes did not significantly affect the weight gain or epididymal, retroperitoneal, and inguinal fat masses in CNO compared to saline-treated mice before CSDS (Additional file 1: Fig. S7B). The SI ratio was similar between CNO mice and saline-treated mice fed the ND that did not experience CSDS. No difference was observed in the anxiety- or depression-like behavioral tests, including the open field, elevated plus-maze, tail suspension, forced swimming, and sucrose preference tests (Additional file 1: Fig. S57C–F).

After the HFD and CSDS, the mRNA levels of the markers expressed by the different astrocyte phenotypes were tested; the activating phenotypes were inhibited, as shown by the mRNA levels of the characteristic markers (Fig. 6B). The mPFC GFAP + cells were immunostained, and the spreading area increased in CNO compared to saline-treated mice after CSDS; a decreasing trend in the number of intersecting astrocytes in CNO mice was also seen (Fig. 6C). Consistent with the change in morphology, astrocyte secretions, including D-serine, glutamate, ATP, and GABA were detected, and the D-serine and glutamate concentrations decreased in CNO compared to saline-treated mice after CSDS (Fig. 6D).

**Fig. 7:**
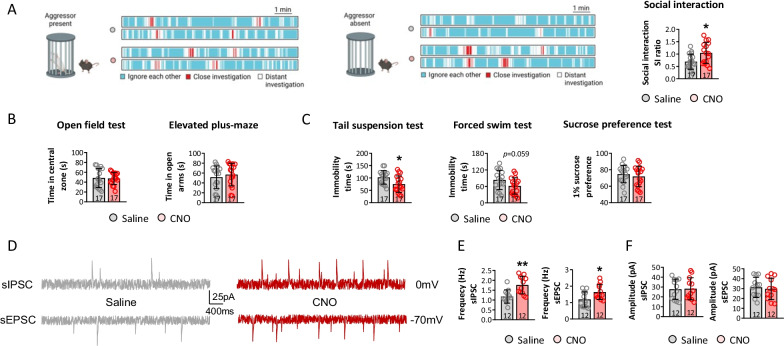
Suppressing astrocytes in mPFC of susceptible mice reversed depressive-like behaviors in HFD mice. **A** To assess the behavioral changes among different mice model, social interaction was performed under aggressor present and absent. **B** The anxiety behaviors of mice were evaluated by open-field test and elevated plus-maze, and **C** depressive behaviors of mice were investigated by tail suspension test, forced swim test, and sucrose preference test. To detect neuronal activities in mPFC, sIPSC and sEPSC (**D**) were recorded, and frequency and amplitude were calculated (**E** and **F**). (*p < 0.05, **p < 0.01 compared with saline-treated mice)

Social behavior was examined in CNO and saline-treated mice to confirm the role of astrocytes in obesity-related depression-like behavior and reduced neuronal activity. The SI ratio increased in CNO compared to saline-treated mice, suggesting that inhibiting astrocytes preserved the decline in social functioning associated with obesity (Fig. 7A). The data from the open field and elevated plus-maze tests revealed no difference between the CNO and saline-treated mice after CSDS, indicating that inhibiting astrocytes did not affect anxiety-like behavior in HFD mice experiencing CSDS (Fig. 7B). Depressive-like behaviors were assessed by the tail suspension, forced swimming, and sucrose preference tests. Immobility times on the tail suspension and forced swimming tests decreased in CNO compared to saline-treated mice, while no difference was observed between CNO and saline-treated mice (Fig. 7C). The sIPSCs and sEPSCs of mPFC neurons were tested in CNO- and saline-treated mice, and the frequencies of sIPSCs and sEPSCs increased in the former, although the amplitudes of the sIPSCs and sEPSCs did not vary significantly between the two groups (Fig. 7D, E and F). These findings suggest that increased reactivation of astrocytes contributed to the depressive-like behaviors induced by CSDS in HFD mice by decreasing neuronal activities in the mPFC.

### JNK–STAT3 signaling involves in regulating astrocytes reactivation in HFD mice

**Fig. 8:**
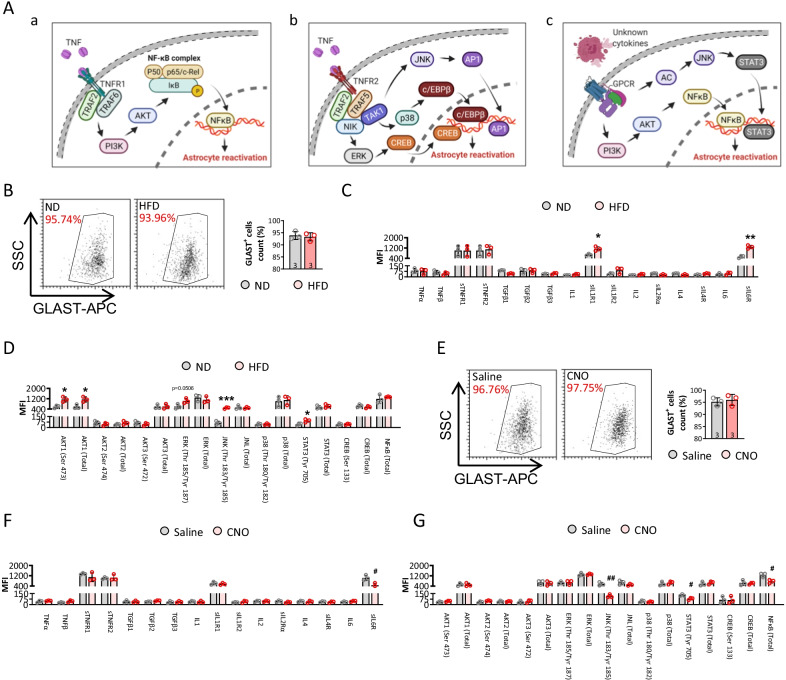
JNK–STAT3 signaling contributes to HFD-induced astrocytes reactivation. **A** Graphic illustration of the signaling pathway in astrocytes related to reactivation. **B** Astrocytes from ND and HFD mice were isolated by MACS and detected by flow cytometry. The astrocytes reactivation related membrane receptors (**C**) and intracellular modulators (**D**) were detected by Milliplex assay. **E** Astrocytes from saline and CNO-injected mice were isolated by MACS and detected by flow cytometry. The astrocytes reactivation related membrane receptors (**F**) and intracellular modulators (**G**) were detected by Milliplex assay. (*p < 0.05, ***p < 0.001 compared with ND mice; ^#^*p* < 0.05, ^##^*p* < 0.001 compared with saline-treated mice)

Astrocytes could sense environmental signals quickly by a wide variety of membrane or intracellular receptors, transporter and ions channels, such as the two well-known TNFR (Fig. 8A-a and b) and GPCR receptors (Fig. 8A–c) [14]. To investigate the exact transcellular and intracellular mechanisms underlying the astrocytes reactivation in HFD mice, we detected multiple related protein level in isolated astrocytes from ND and HFD mice (Fig. 8B–D). Notably, IL1R, IL6R, pAKT1, pJNK and pSTAT3 were dramatically upregulated post high-fat diet. On contrast, pJNK, pSTAT3, and NFκB were significantly depressed in HFD mice when astrocytes were deactivated (Fig. 8E–G). These results highlighted the important role of JNK–STAT3 signaling in modulating astrocytes reactivation during high-fat diet. However, the JNK–STAT3 pathway need to be further confirmed of their roles and function in astrocytes reactivation by genetic knockout and overexpression assay; and more related receptors and intracellular modulators need to be investigated in the future studies.

## Discussion

The present series of experiments showed that an HFD and leptin knockout increased body and adipose mass, and induced depressive-like behaviors after CSDS. The data suggest that the HFD and leptin knockout following CSDS produced depressive-like behaviors by priming astrocytic reactivation in the mPFC, which increased levels of D-serine and glutamate related to inhibitory neuronal presynaptic activities in the mPFC. The effect of reactivating astrocytes on depressive-like behaviors and neuronal activities in obese mice subjected to CSDS was validated using GfaABC1D-hM3Dq-AAV or GfaABC1D-hM3Dq-AAV injections. The findings suggest that reactivation of astrocytes promoted depressive-like behaviors, in association with a decline of neuronal activity in obese CSDS-susceptible mice.

Although clinical and preclinical studies have shown that obesity is linked to behavioral and physiological changes, results vary and comparisons between studies are complicated by inconsistencies in the definitions of exposure and outcome, as well as in study populations and animal models [47–50]. Our obese animal model was constructed by feeding mice with high-fat food for 8 weeks, or by leptin knockout, both of which increased BW and abdominal adiposity (corroborated by increased epididymal, retroperitoneal, and inguinal fat mass). The results from this study confirm that leptin knockout increases depressive-like behaviors; there was a tendency toward depression in the obese animal model. Positive correlations have been found between obesity and low self-esteem and body dissatisfaction in cross-sectional studies of children and adolescents, which may be the underlying mechanism of psychological disorders such as depression and anxiety [51]. It is well known that depression is accompanied by deficits in social functioning [52]. Our results showed no variations in social functioning or anxiety-like behaviors between obese and normal-weight mice not subjected to CSDS. The increase in immobility time on the tail suspension test suggested that obesity increased the tendency toward depression rather than directly inducing a psychological disorder in the absence of CSDS.

Stress precipitates psychiatric disease, particularly in vulnerable individuals, such as those with major depressive disorder and post-traumatic stress disorder, while exercise and chronic blockade of stress hormones increase stress resilience in mice [53]. CSDS is commonly utilized in animal models of depression. Social defeat stress promotes depression via synaptic structural changes in the PFC and amygdala [54], which affect the maturation of newly generated neurons in the hippocampus [55] and induce neurovascular pathology [56]. In the current study, obese mice experiencing CSDS showed a significant decline in social functioning and depressive-like behaviors, similar to previous reports [57, 58]. In this study, obesity per se did not induce depression, but increased the risk thereof under social stress.

Chronic stress and depressive-like behaviors in basic neuroscience research have been associated with impaired neuroplasticity, such as neuronal atrophy and synaptic loss in the mPFC, which participates in social cognition and emotion regulation. Moreover, the hippocampus is involved in learning and memory [59]. The frequency of spontaneous synaptic activity in the mPFC was reduced in obese compared to normal-weight animals after CSDS in this study, suggesting a decrease in synaptic functioning due to obesity and chronic stress. Chronic stress results in sustained decreases in the expression and signaling of brain-derived neurotrophic factors, and in the number and function of synapses in the mPFC and hippocampus [60, 61]. Furthermore, dendritic spine density decreased in the mPFC in response to obesity and CSDS compared to our normal-weight mice. In addition, there was no change in the proportion of mushroom-shaped spines, which are functional dendritic spines; this may underlie the depressive-like behaviors seen in obese mice after CSDS. Some therapies for clinical depression aim to decrease activity in the amygdala in response to threat cues and attenuate the exacerbation of depressive symptomology and maladaptive problem-solving behavior associated with amygdala hyperactivation [62]. Neurocircuits from the mPFC directly project to inhibitory interneurons in the amygdala that regulate fear reactions and anxiety [63]. Obesity did not markedly reduce spontaneous synaptic activity in the hippocampus or amygdala in our study, reflecting the lack of a difference in anxiety behavior between obese and normal-weight animals after CSDS.

Microglia and astrocytes are found throughout the brain, and are highly elaborate and plastic as a result of arborization [64, 65]. HFD-induced obesity drives the accumulation and proliferation of activated microglia and astrocytes seen in the hypothalamic region [66, 67]. Metabolic changes in obese mice rapidly alter the expression of the leptin receptor responsible for leptin-induced calcium signaling in astrocytes, and astrocytic activity in the hypothalamus [68]. However, glial responses to obesity induced by a high-energy-dense diet were not examined in this study. We showed that obesity promoted the A1-specific phenotype, which weakened the blood–brain barrier and pruned synapses in the mPFC, even though the number of astrocytes in obese mice did not differ from that in normal-weight mice [22]. Neuroprotective A2-specific astrocytes, characterized by specific markers, did not change in obese mice, and both M1 and M2 (M2a, M2b, and M2c) microglia were similar in obese and normal-weight mice after CSDS. There is evidence that crosstalk between microglia and astrocytes participates in the pathological process of depression [46]. In this study, no microglia were activated in the obese or CSDS mice, but reactivation of astrocytes was reflected by changes in astrocyte morphology, including a larger spreading area and more intersecting astrocytes in obese compared to normal-weight mice. Astroglia are secretory cells that release neuroactive molecules, including glutamine, ATP, and classical neurotransmitters, to modulate neuronal activity and synaptic transmission [69]. In this study, the release of D-serine and glutamate by astrocytes, as measured in the microdialysate, was increased in obese compared to normal-weight mice, indicating reactivation of astrocytes.

Besides neurons, astrocytes are presently viewed as an important source of excitable cells on operating neuronal networks in CNS. In response to HFD-induced neuroinflammation and saturated free fatty acids [19], astrocytes generate excitation through activating Ins (1,4,5) P3 receptor (IP3R)-triggered Ca2 + release from the endoplasmic reticulum and then deliver feedback or feedforward messages to modulate neighboring cells function (such as synaptic transmission, neuronal synchronization, and cerebral flow) via releasing gliotransmitters. In particular, astrocytes facilitate excitatory synaptic transmission via releasing glutamate (activating mGluRs) [10] and D-serine (activating NMDA receptors) [11] while inhibit excitatory synaptic transmission with adenosine (inhibiting A1-adenosine receptors) [12, 13]; astrocytes also control inhibitory synaptic transmission through feedback release of glutamate and ATP [14, 15]. Brain pathological conditions, favor multiple gliotransmitters release from astrocytes, which exacerbated neuronal toxicity induced by neuron-derived glutamate over-stimulation and inhibited synaptic transmission [16, 17].

Genetically identical mice can display phenotypic differences after exposure to chronic stress, and phenotypic variability in inbred mice has been attributed to environmental factors, such as variations in prenatal and postnatal development and dominance hierarchies [70]. Susceptibility to stress plays a vital role in affective dysregulation, such as stress-induced depressive-like traits, which are associated with dysregulation of the immune system and structural changes in limbic brain areas such as the mPFC [71]. Regarding the deficits in SI and depressive-like behaviors seen in this study after consuming the HFD and exposure to CSDS, the resilient mice were characterized by an SI ratio ≥ 1 and immobility time < 100 s, while the susceptible mice had an SI ratio < 1 and immobility time ≥ 100 s. Astrocytic reactivation, which manifests as elevated neurotoxic A1 levels, contributes to depressive-like behavior and memory deficits [72], and increases the size of the spreading area, number of intersecting astrocytes, and amount of neuroactive molecules released, including D-serine and glutamate. The increases in BW and epididymal, retroperitoneal, and inguinal fat masses were similar between our resilient and susceptible mice. A decline in social function and enhanced depressive-like behaviors were seen only in susceptible obese mice in response to CSDS stress; synaptic activity also decreased, as in previous studies, which contributed to the behavioral changes.

A previous study showed that obesity affects astrocytes, thus disrupting the delicate balance between excitatory and inhibitory transmission in the PFC [73]. Increased activation of astrocytes causes exocytosis of glutamate and activation of presynaptic NMDA receptors, in turn increasing presynaptic terminal excitability and neurotransmitter release [23]. In our study, the mPFC GfaABC1D-hM3Dq AAV and CNO injections promoted time-restricted activation in HFD mice suffering from CSDS. Reactivation of astrocytes, revealed by the pan-reactive, A1-specific, and A2-specific markers, spreading area, and number of intersecting astrocytes, as well as D-serine, glutamate, and ATP, increased in both resilient and susceptible mice [38]. Moreover, resilient and susceptible mice fed the HFD and subjected to CSDS exhibited synaptic activity in the mPFC, along with similar SIs and affective behaviors. The GfaABC1D-hM4Di AAV injection into the mPFC and i.p. injection of CNO selectively inhibited astrocytic reactivation in HFD mice, as confirmed by gene expression of the Pan-reactive, A1-specific, and A2-specific markers, morphological changes and a decrease in neuroactive molecular activity [74]. Interestingly, while GfaABC1D-hM4Di AAV was injected into the mPFC of HFD mice not subjected to CSDS, the inhibition of astrocytes by i.p. injection of CNO did not change body or adipose tissue weights, SI, or affective behavior compared to the saline-treated mice. However, the significant deficit in SIs and depressive-like behavior were ameliorated in astrocyte-inhibited HFD mice under CSDS, along with an increase in neuronal activity. These results suggest that neuronal activity in the mPFC can be weakened by reactivating astrocytes associated with attenuated SIs and increased depressive-like behaviors, and enhanced by inhibiting astrocytes linked to reinforcement of SIs and depressive-like behaviors. Notably, the mechanisms underlying the signaling pathways remain to be elucidated, and further studies are needed to determine the factors responsible for the reactivation of astrocytes during the pathological processes of obesity. Targets for future studies include elucidating the role of astrocytic reactivation in the tendency for depressive behavior in obese mice, and determining whether there is bidirectional crosstalk between neurons and astrocytes, and whether CSDS-induced depressive behaviors in obese individuals are linked to the neurocircuitry connecting different brain regions.

There still have a lot limits of this paper, especially lacking of exact mechanisms on regulating astrocytes reactivation. It also should be noted that the application of ketamine (even though all the mice were treated the same with ketamine prior to perfusion) would have a rapid effect on spines in the mPFC [75] which could affect the interpretation of the data. 

## Conclusion

Taken together, our results indicate that reactivation of astrocytes in the mPFC of obese mice under CSDS decreased synaptic activity and increased excitation, eventually inducing a deficit in SIs and depressive-like behaviors. These findings improve our understanding of the pathological relationship between obesity and depression.

## Supplementary Information

**Additional file 1: Table S1.** Primers used for qPCR. **Figure S1.** Neither high fat diet nor leptin knockout induce depressive-like behavior. **Figure S2.** Neuronal activities and astrocytes reactivity in hippocampus and amygdala were not altered among ND, HFD, LM, and ob/ob mice. **Figure S3.** E/I balance was not altered by high fat diet or leptin knockout. **Figure S4.** Astrocyte reactivation was significantly upregulated by high fat diet or leptin knockout, but microglia response was not altered. **Figure S5.** Astrocyte reactivity was increased in susceptible mice. **Figure S6.** Depressive-like behaviors were increased in susceptible mice. **Figure S7.** Behavioral functions were not altered before CNO injection.

## Data Availability

The datasets used and/or analyzed details during the current study are available from the corresponding author on reasonable request.

## Abbreviations

AAV: Adeno-associated viruses
aCSF: Artificial cerebrospinal fluid
ATP: Adenosine triphosphate
BSA: Bovine serum albumin
BW: Body weight
CNO: Clozapine-N-oxide
CNS: Central nervous system
CSDS: Chronic social defeat stress
GFAP: Glial fibrillary acidic protein
GLAST: Glutamate-aspartate transporter
HFD: High-fat diet
i.p.: Intraperitoneal
LM: Littermate
mEPSCs: Miniature excitatory postsynaptic currents
mIPSCs: Miniature inhibitory postsynaptic currents
mPFC: Medial prefrontal cortex
ND: Normal diet
NMDA: N-Methyl-d-aspartic acid receptor
ob/ob mice: Leptin-deficient mice
PBS: Phosphate-buffered saline
PFA: Paraformaldehyde
qRT-PCR: Quantitative real-time polymerase chain reaction
sEPSCs: Spontaneous excitatory postsynaptic currents
SI: Social interaction
sIPSCs: Spontaneous inhibitory postsynaptic currents
TST: Tail suspension test
TTX: Tetrodotoxin
