# Supplementary material for: Astrocyte reactivation in medial prefrontal cortex contributes to obesity-promoted depressive-like behaviors
Source: J Neuroinflammation. 2022 Jun 27;19:166. doi: 10.1186/s12974-022-02529-4 (PMC9235218; doi:10.1186/s12974-022-02529-4)
Supplement: Supplementary file 1 — Additional file 1: Table S1. Primers used for qPCR. Figure S1. Neither high fat diet nor leptin knockout induce depressive-like behavior. Figure S2. Neuronal activities and astrocytes reactivity in hippocampus and amygdala were not altered among ND, HFD, LM, and ob/ob mice. Figure S3. E/I balance was not altered by high fat diet or leptin knockout. Figure S4. Astrocyte reactivation was significantly upregulated by high fat diet or leptin knockout, but microglia response was not altered. Figure S5. Astrocyte reactivity was increased in susceptible mice. Figure S6. Depressive-like behaviors were increased in susceptible mice. Figure S7. Behavioral functions were not altered before CNO injection. [file 12974_2022_2529_MOESM1_ESM.docx]

**Supplementary materials**

**Title: Astrocyte reactivation in medial prefrontal cortex contributes to obesity promoted depressive-like behaviors**

Authors: Gang Yu, et al.

Contents: Table S1, Figure S1-7

Table S1. Primers used for qPCR

| **Gene** | **Forward primer (5'-3')** | **Reverse primer (5'-3')** |
| --- | --- | --- |
| *Leptin* | GTGGCTTTGGTCCTATCTGTC | CGTGTGTGAAATGTCATTGATCC |
| *Il1b* | GAAATGCCACCTTTTGACAGTG | TGGATGCTCTCATCAGGACAG |
| *Il6* | CTGCAAGAGACTTCCATCCAG | AGTGGTATAGACAGGTCTGTTGG |
| *Il12a* | AGACATCACACGGGACCAAAC | CCAGGCAACTCTCGTTCTTGT |
| *Tnf* | CAGGCGGTGCCTATGTCTC | CGATCACCCCGAAGTTCAGTAG |
| *Ifng* | ATGAACGCTACACACTGCATC | CCATCCTTTTGCCAGTTCCTC |
| *Ccl5* | TTTGCCTACCTCTCCCTCG | CGACTGCAAGATTGGAGCACT |
| *Ccl20* | GCCTCTCGTACATACAGACGC | CCAGTTCTGCTTTGGATCAGC |
| *Cxcl1* | CTGGGATTCACCTCAAGAACATC | CAGGGTCAAGGCAAGCCTC |
| *Cxcl10* | TGCAAGTCTATCCTGTCCGC | ACGGAGCTCTTTTTGACCTTC |
| *Csf2* | GGCCTTGGAAGCATGTAGAGG | GGAGAACTCGTTAGAGACGACTT |
| *Cd16* | AATGCACACTCTGGAAGCCAA | CACTCTGCCTGTCTGCAAAAG |
| *Cd86* | TCAATGGGACTGCATATCTGCC | GCCAAAATACTACCAGCTCACT |
| *Nos2* | GTTCTCAGCCCAACAATACAAGA | GTGGACGGGTCGATGTCAC |
| *Il1ra* | GCTCATTGCTGGGTACTTACAA | CCAGACTTGGCACAAGACAGG |
| *Il4* | GGTCTCAACCCCCAGCTAGT | GCCGATGATCTCTCTCAAGTGAT |
| *Tgfb1* | CCACCTGCAAGACCATCGAC | CTGGCGAGCCTTAGTTTGGAC |
| *Ccl22* | ATGCGCGTCCATTACCTGTG | TCAACGGTCCAATCATTTGCT |
| *Cd206* | CTCTGTTCAGCTATTGGACGC | TGGCACTCCCAAACATAATTTGA |
| *Cd163* | GGTGGACACAGAATGGTTCTTC | CCAGGAGCGTTAGTGACAGC |
| *Arg1* | CTCCAAGCCAAAGTCCTTAGAG | GGAGCTGTCATTAGGGACATCA |
| *Ym1* | CAGGTCTGGCAATTCTTCTGAA | GTCTTGCTCATGTGTGTAAGTGA |
| *Fizz1* | CCAATCCAGCTAACTATCCCTCC | ACCCAGTAGCAGTCATCCCA |
| *Il10* | CTTACTGACTGGCATGAGGATCA | GCAGCTCTAGGAGCATGTGG |
| *Il4ra* | ACACTACAGGCTGATGTTCTTCG | TGGACCGGCCTATTCATTTCC |
| *Csf3* | ATGGCTCAACTTTCTGCCCAG | CTGACAGTGACCAGGGGAAC |
| *Lcn2* | CCGACACTGACTACGACCAG | AATGCATTGGTCGGTGGGAA |
| *Steap4* | CAAACGCCGAGTACCTTGCT | CAGACAAACACCTGCCGACT |
| *S1pr3* | CTTGCAGAACGAGAGCCTGT | CCTCAACAGTCCACGAGAGG |
| *Timp1* | CGCTAGAGCAGATACCACGA | CCAGGTCCGAGTTGCAGAAA |
| *Hspb1* | GAGATCACTGGCAAGCACGA | ATTGTGTGACTGCTTTGGGC |
| *Cxcl10* | TGCAAGTCTATCCTGTCCGC | ACGGAGCTCTTTTTGACCTTC |
| *Cd44* | TCAGGATAGCCCCACAACAAC | GACTCCGTACCAGGCATCTTC |
| *Osmr* | GTCATTCTGGACATGAAGAGGT | AATCACAGCGTTGGGTCTGA |
| *Cp* | GATGTTTCCCCAAACGCCTG | GTAGCTCTGAGACGATGCTTGA |
| *Serpina3n* | GTCTTTCAGGTGGTCCACAAGG | GCCAATCACAGCATAGAAGCG |
| *Aspg* | CAGGTGCCCAGGTTCCTATC | GTCCACCTTGGTTGTCCGAT |
| *Vim* | GAGGAGATGAGGGAGTTGCG | CTGCAATTTTTCTCGCAGCC |
| *Gfap* | AACCGCATCACCATTCCTGT | TCCTTAATGACCTCGCCATCC |
| *H2.T23* | ATTGGAGCTGTTGTGAGGAGG | CCACGAGGCAACTGTCTTTTC |
| *Serping1* | TGGCTCAGAGGCTAACTGGC | GAATCTGAGAAGGCTCTATCCCCA |
| *H2.D1* | ATGGAACCTTCCAGAAGTGGG | GAAGTAAGTTGGAGTCGGTGGA |
| *Ggta1* | TCTCAGGATCTGGGAGTTGGA | GAGTTCTATGGAGCTCCCGC |
| *Iigp1* | ATTTGGCTCGAAGCCTTTGC | ACGGCATTTGCCAGTCCTTA |
| *Gbp2* | TAAAGGTCCGAGGCCCAAAC | AACATATGTGGCTGGGCGAA |
| *Fbln5* | AGGGGGTTAAGCGAAACCAG | GTGAGTATCCTTTTAATCCTGGCA |
| *Ugt1a* | GGAAGCTGTTAGTGATCCCC | TGCTATGACCACCACTTCGT |
| *Fkbp5* | TGCAGTGTCGGCAGTTGTAT | GGGTCGCCCAAGTTAGAACA |
| *Psmb8* | TATCTGCGGAATGGGGAACG | AAAGTCCCGGTCCCTTCTTG |
| *Srgn* | GTTCAAGGTTATCCTGCTCGGA | AAACAGGATCGGTCATCGGG |
| *Amigo2* | GTTCGCCACAACAACATCAC | GTTTCTGCAAGTGGGAGAGC |
| *Clcf1* | GACTCGTGGGGGATGTTAGC | CCCCAGGTAGTTCAGGTAGGT |
| *Tgm1* | AGACCCAATTTTCCTGGGGC | AGCGAGGACCTTCCATTGTG |
| *Ptx3* | CATCCCGTTCAGGCTTTGGA | CACAGGGAAAGAAGCGAGGT |
| *S100a10* | GAAAGGGAGTTCCCTGGGTT | CCCACTTTTCCATCTCGGCA |
| *Sphk1* | AAAGCGAGACCCTGTTCCAG | CAGTCTGCTGGTTGCATAGC |
| *Cd109* | GTCGCTCACAGGTACCTCAA | CTGTGAAGTTGAGCGTTGGC |
| *Ptgs2* | CTCAGCCATGCAGCAAATCC | GGGTGGGCTTCAGCAGTAAT |
| *Emp1* | ACCATTGCCAACGTCTGGAT | TGGAACACGAAGACCACGAG |
| *Slc10a6* | TCCATAGAGACCGGAGCACA | ATGCCTGATATGCTGCGACA |
| *Tm4sf1* | CTGAGGGACAGTACCTTCTGGATTC | GGCTAGGCCTCAACACAGTTA |
| *B3gnt5* | TGCTCCTGGATGAAAGGTCC | ACATGCTTGATCCGTGTGGT |
| *Cd14* | TCAGAATCTACCGACCATGAAGC | GGACACTTTCCTCGTCCTGG |
| *Gapdh* | AGGTCGGTGTGAACGGATTTG | GGGGTCGTTGATGGCAACA |


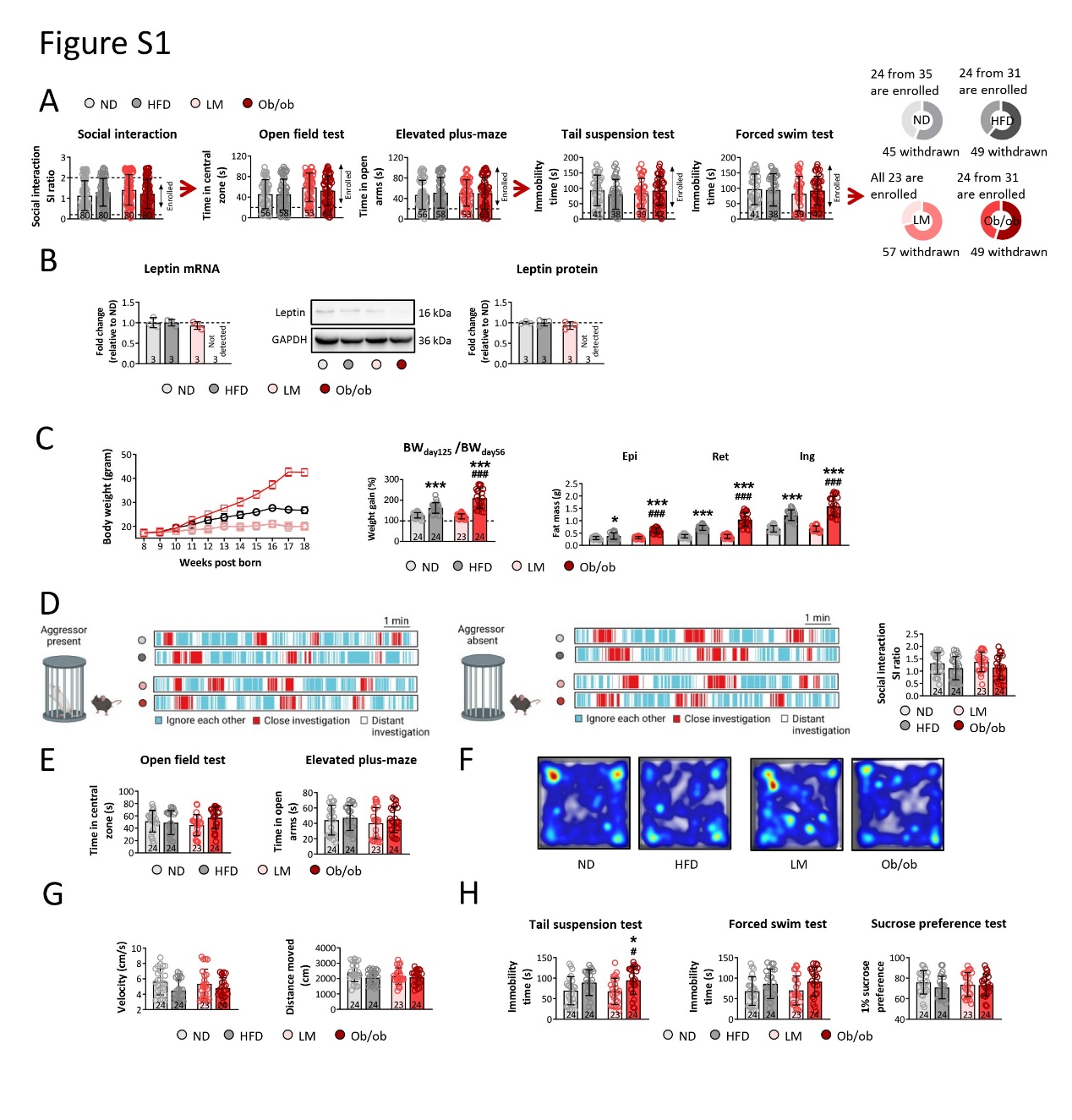


**Neither high fat diet nor leptin knockout induce depressive-like behavior.** (A) To avoid the bias induced by individual differences, the mice used for further study were screened by social interaction, open field test, elevated plus-maze, tail suspension test, and forced swim test. (B) The mRNA and protein level were detected in the brain of different mice model. (C) The body weight was recorded, weight gain was measured by dividing 125-day body weight to 56-day body weight, and fat mass was measured in epididymal, inguinal, and retroperitoneal regions. (D) To assess the behavioral changes among different mice model, social interaction was performed under aggressor present and absent. (E) The anxiety behaviors of mice were evaluated by open field test and elevated plus-maze, (F and G) the locomotor activity was evaluated by analyzing velocity and distance moved on open field test, and (H) depressive behaviors of mice were investigated by tail suspension test, forced swim test, and sucrose preference test. (ND represented as normal diet, HFD represented as high fat diet, LM represented as littermate mice, and ob/ob represented as obesity transgenic mice; data is presented as mean ± SD, *p<0.05, ***p<0.001 compared with ND mice; ^###^p<0.001 compared with LM mice).


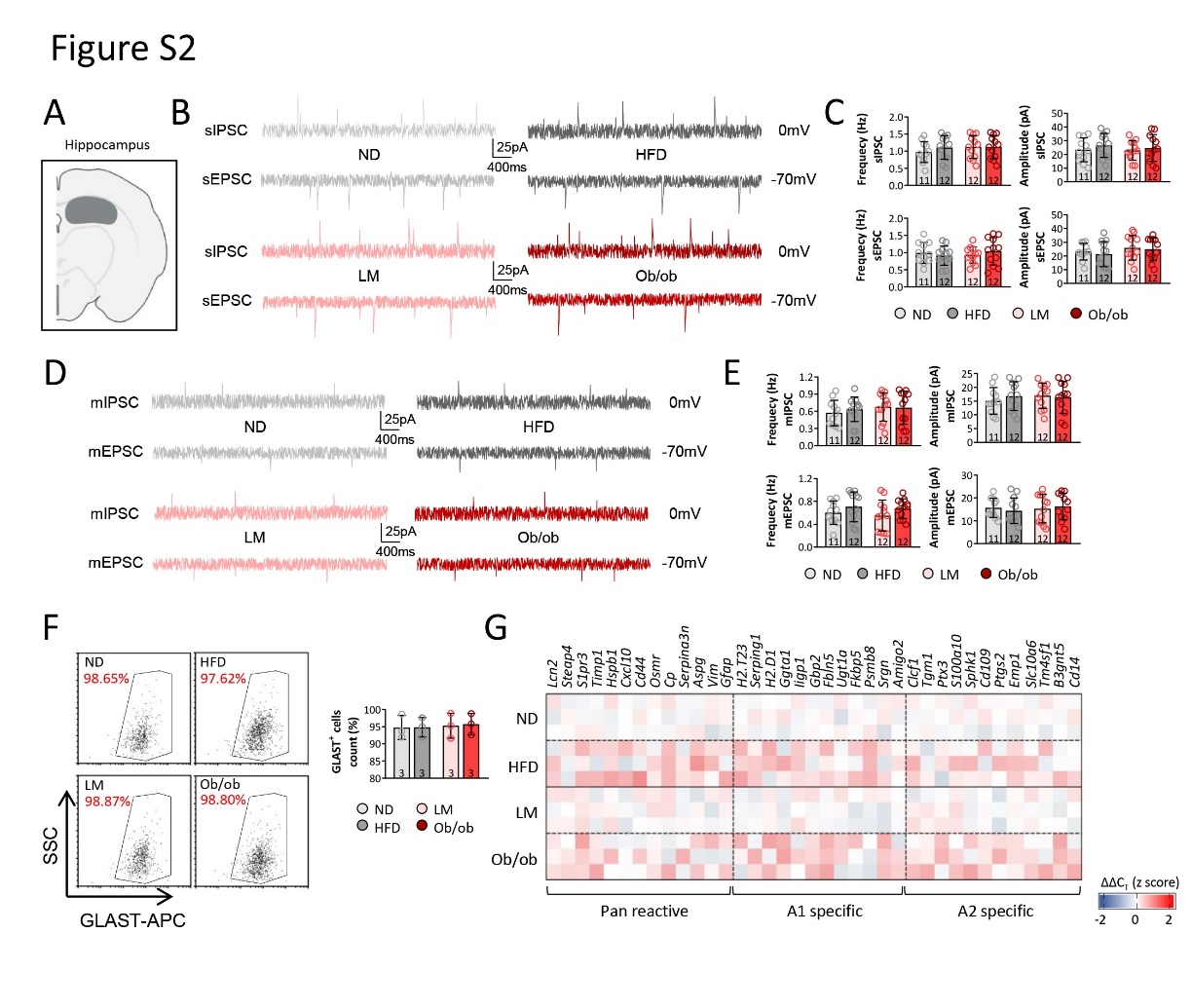


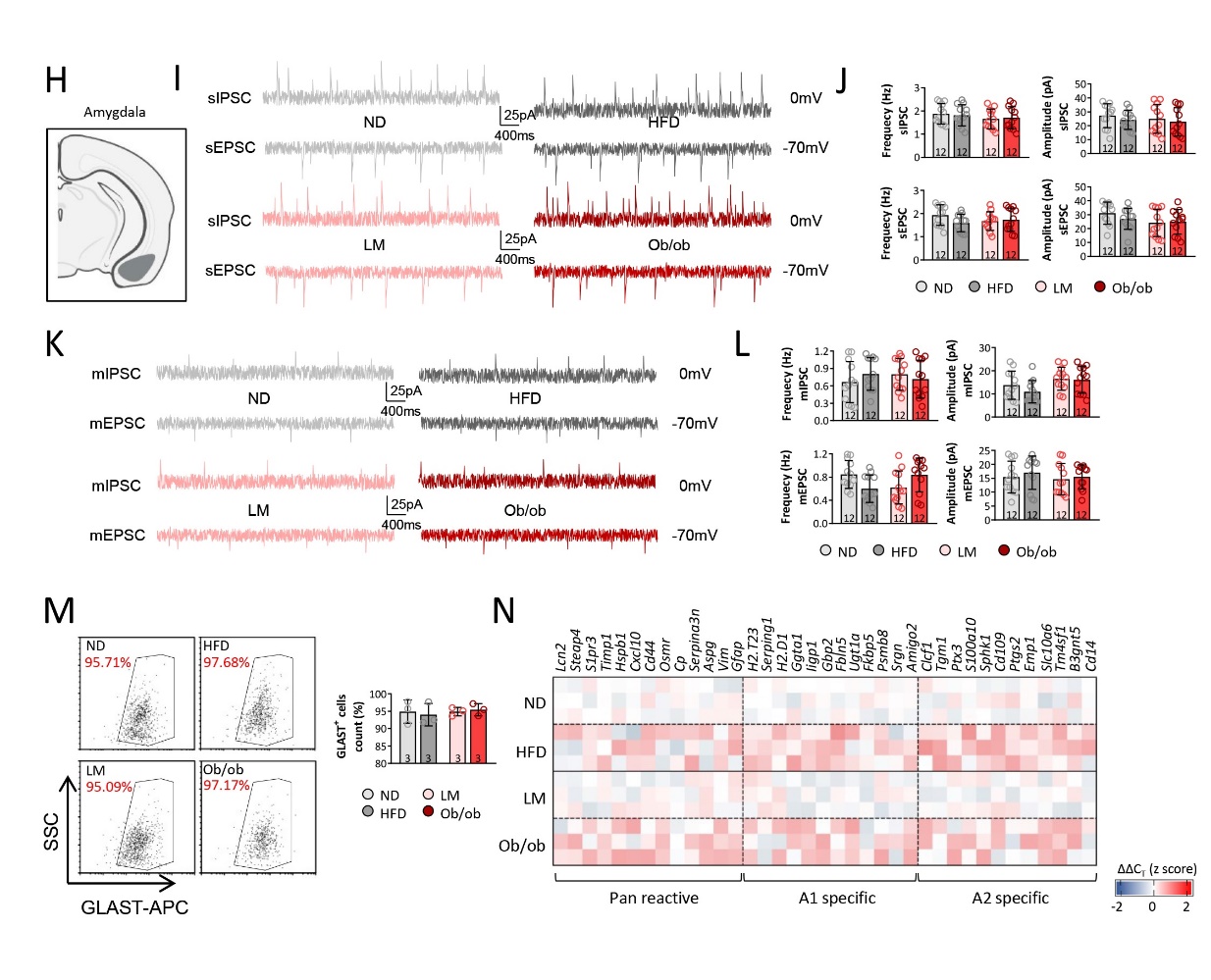


**Neuronal activities and astrocytes reactivity in hippocampus and amygdala were not altered among ND, HFD, LM, and ob/ob mice.** To detected neuronal activities in hippocampus (A), sIPSC, sEPSC (B, C), mIPSC, and mEPSC (D, E) were recorded, and frequency and amplitude were calculated. Astrocyte response in hippocampus was detected by investigating activation related genes expression of isolated astrocyte (F and G). To detected neuronal activities in amygdala (H), sIPSC, sEPSC (I, J), mIPSC, and mEPSC (K, l) were recorded, and frequency and amplitude were calculated. Astrocyte response in amygdala was detected by investigating activation related genes expression of isolated astrocyte (M and N). (Data is presented as mean ± SD, p<0.05 was considered as significant difference).


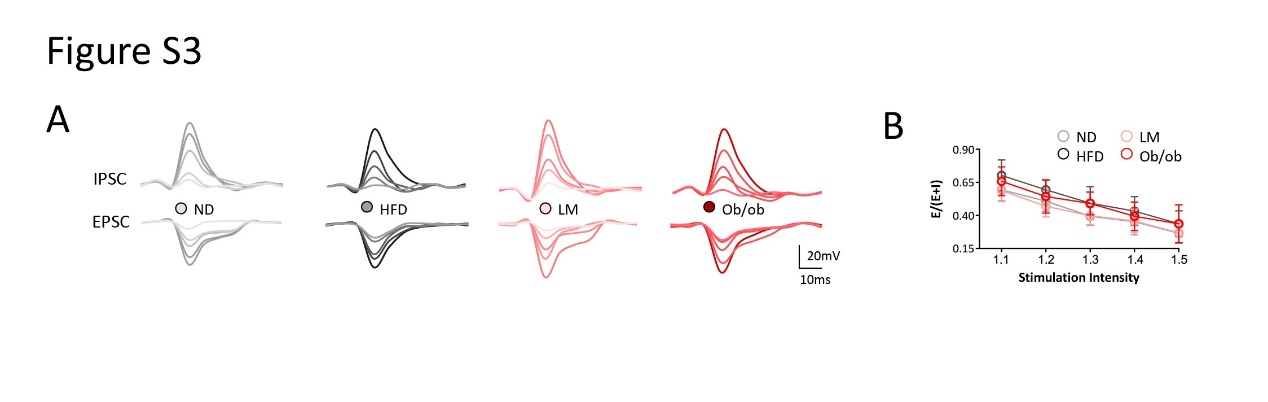


**E/I balance was not altered by high fat diet or leptin knockout.** (A) IPSC and EPSC was recorded in mPFC brain slices and (B) E/I balance was calculated by E/(E+I). (Data is presented as mean ± SD, p<0.05 was considered as significant difference)


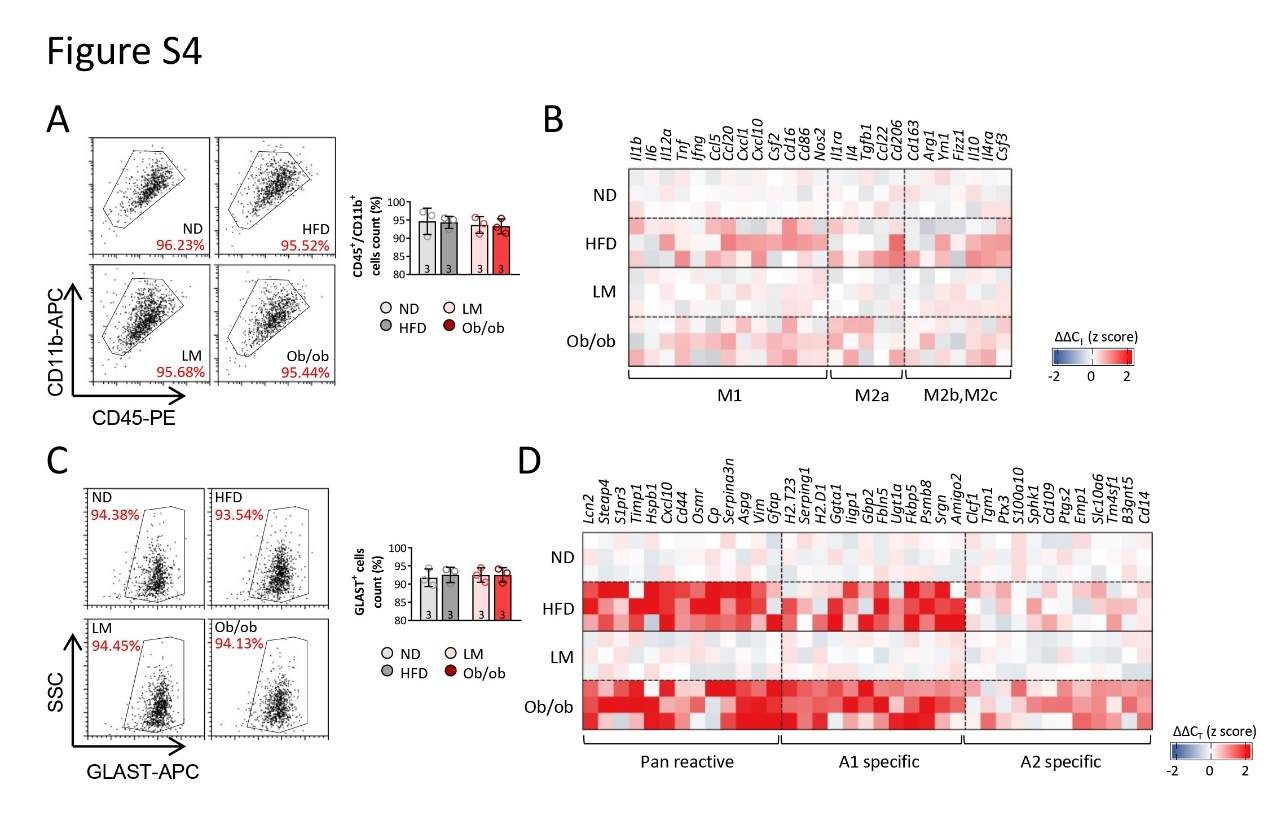


**Astrocyte reactivation was significantly upregulated by high fat diet or leptin knockout, but microglia response was not altered.** Microglia response was detected by investigating activation related genes expression of isolated microglia (A and B). Astrocyte response was detected by investigating activation related genes expression of isolated astrocyte (C and D). (Data is presented as mean ± SD, p<0.05 was considered as significant difference).


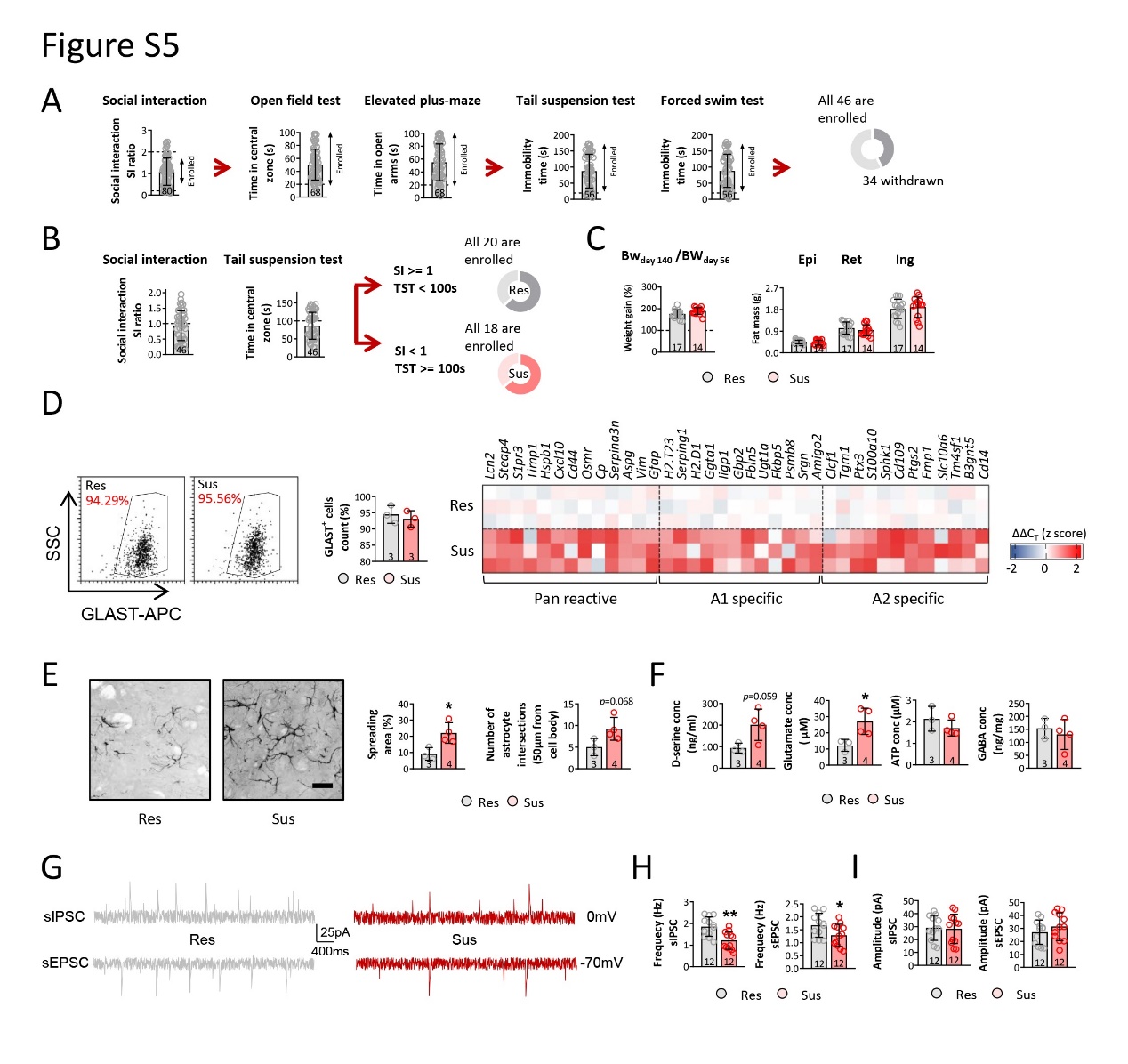


**Astrocyte reactivity was increased in susceptible mice.** (A) To avoid the bias induced by individual differences, the mice used for further study were screened by social interaction, open field test, elevated plus-maze, tail suspension test, and forced swim test. (B) The resilient mice were defined as SI >= 1 and TST < 100s while susceptible mice were defined as SI < 1 and TST >= 100s. (C) Fat mass was measured in epididymal, inguinal, and retroperitoneal regions. (D) Astrocyte response was detected by investigating activation related genes expression of isolated astrocyte. (E) Astrocyte morphology was measured by calculating spreading area and number of interactions. (F) The level of D-serine, glutamate, ATP, and GABA were also assessed in microdialysate from mPFC. To detected neuronal activities in mPFC, sIPSC and sEPSC were recorded (G), and frequency and amplitude were calculated (H and I). (Res represented as resilient mice, Sus represented as susceptible mice; data is presented as mean ± SD, *p<0.05, **p<0.01 compared with Res mice).


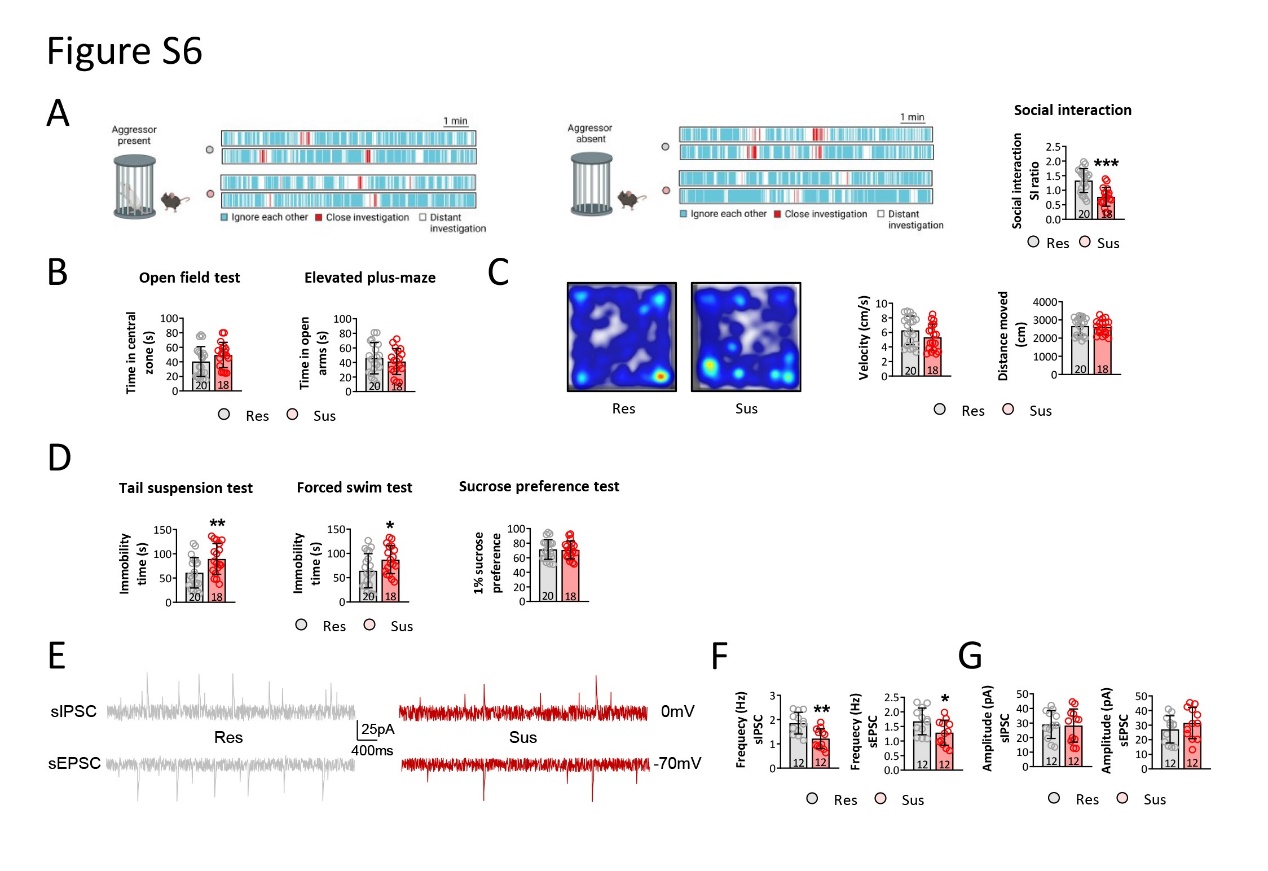


**Depressive-like behaviors were increased in susceptible mice.** (A) To assess the behavioral changes among different mice model, social interaction was performed under aggressor present and absent. (B) The anxiety behaviors of mice were evaluated by open field test and elevated plus-maze, (C) the locomotor activity was evaluated by analyzing velocity and distance moved on open field test, and (D) depressive behaviors of mice were investigated by tail suspension test, forced swim test, and sucrose preference test. To detected neuronal activities in mPFC, sIPSC and sEPSC were recorded (E), and frequency and amplitude were calculated (F and G). (Res represented as resilient mice, Sus represented as susceptible mice; data is presented as mean ± SD, *p<0.05, **p<0.01, ***p<0.001 compared with Res mice).


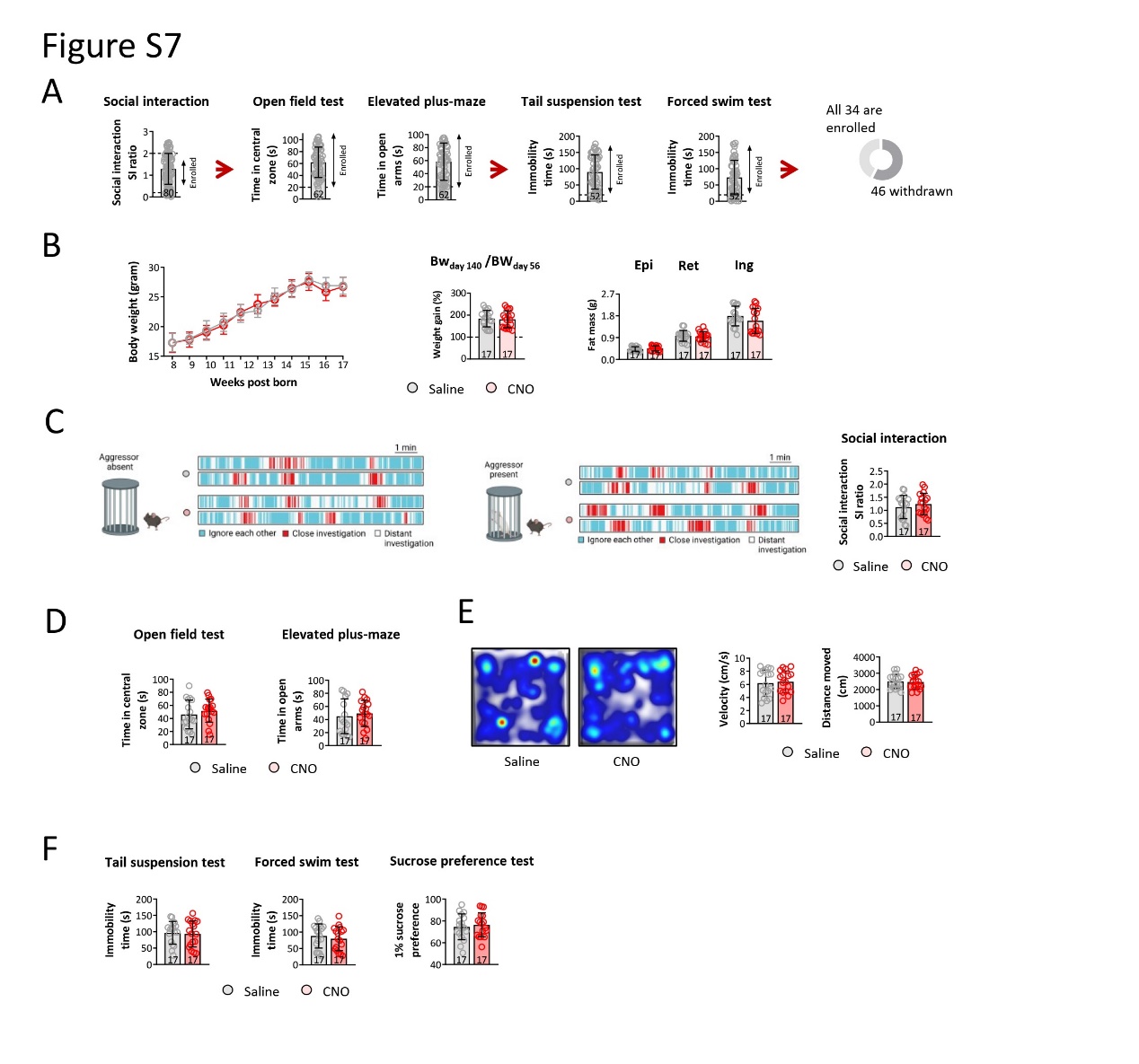


**Behavioral functions were not altered before CNO injection.** (A) To avoid the bias induced by individual differences, the mice used for further study were screened by social interaction, open field test, elevated plus-maze, tail suspension test, and forced swim test. (B) The body weight was recorded, weight gain was measured by dividing 125-day body weight to 56-day body weight, and fat mass was measured in epididymal, inguinal, and retroperitoneal regions. (C) To assess the behavioral changes among different mice model, social interaction was performed under aggressor present and absent. (D) The anxiety behaviors of mice were evaluated by open field test and elevated plus-maze, (E) the locomotor activity was evaluated by analyzing velocity and distance moved on open field test, and (F) depressive behaviors of mice were investigated by tail suspension test, forced swim test, and sucrose preference test. (Data is presented as mean ± SD, p<0.05 was considered as significant difference).
